# Systematic Evaluation of Different Ribonucleoprotein Complexes as Posttranscriptional Biosensors in Cell-Free TX-TL Systems

**DOI:** 10.1021/acssynbio.6c00170

**Published:** 2026-05-05

**Authors:** Dudu Boyvat, Francesco Grassi, Lucia Cassella, Velia Siciliano

**Affiliations:** Istituto Italiano di Tecnologia-IIT, 378791 Synthetic and Systems Biology lab for Biomedicine, Largo Barsanti e Matteucci 53, 80125 Naples, Italy

## Abstract

Synthetic biology has enabled the development of sophisticated cell-free biosensors capable of complex, sensitive signal detection with promising clinical and environmental applications due to their ease of use, fast readout development, portability, and built-in biosafety. Nevertheless, the potential of ribonucleoprotein (RNP) complexes as posttranscriptional biosensors in cell-free transcription-translation (TX-TL) systems remains largely unexplored. In this study, we test the performance of three different posttranscriptional circuits (RISC:*miRNA*, MS2-CNOT7:*ms2L*, and L7Ae:*k-turn*) in different TX-TL cell-free platforms (eukaryotic, prokaryotic, and recombinant) and evaluate the robustness of their reconstitution in vitro. We find that miRNA sensors encoded in in vitro-transcribed (IVT) luciferase RNAs work effectively, yet the difficulty in reliably quantifying the basal expression levels of the reporter gene represents a major bottleneck of IVT RNA-based miRNA sensors. Although the MS2-CNOT7:*ms2L* RNP circuit was robustly reconstituted in eukaryotic lysates, we found that lysate-dependent constraints hamper its use as an in vitro biosensor, reducing its programmability potential in the cell-free context. On the other hand, L7Ae:*k-turn* was successfully reconstituted in all cell-free platforms tested following thorough characterization of plasmid-specific and protocol-dependent effects on reporter expression and its conditional repression by L7Ae. Finally, we successfully introduced an additional control layer by implementing protease-responsive L7Ae-mediated conditional repression in the PURE system, indicating the potential of the L7Ae:*k-turn* RBP circuit as a promising cell-free biosensor for protease-based viral detection in vitro.

## Graphical abstract

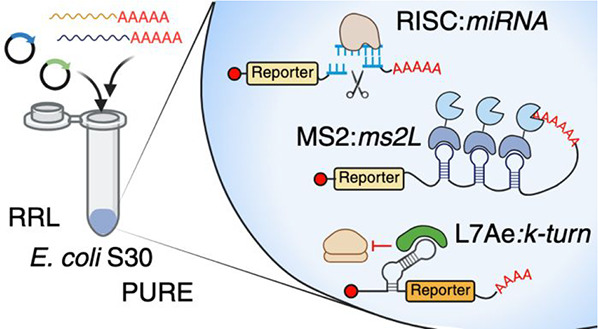

## Introduction

In the last years, cell-free systems (CFSs) have swerved from answering fundamental biological questions to a protein synthesis platform that could be harnessed for biomanufacturing, biological parts prototyping, and nucleic acid detection. One potential application of CFS lies in developing sensing platforms programmed to conditionally express a reporter protein upon target analyte addition. Cell-free biosensors have recently gained attention for their ease of use, fast readout development, portability, and built-in biosafety.^1^ Further, synthetic biology has enabled the development of sophisticated biosensors capable of complex and sensitive signal detection by incorporating Boolean logic-based genetic circuits with engineered components to process a variety of environmental signals into a measurable output. Such biotechnological advances have laid the foundations for the sensitive and accurate detection of viral RNA,^2–6^ gut microbiota,^7^ or protein biomarkers^8^ toward promising clinical applications. To date, most of the synthetic biology-based “smart” biosensors comprise riboswitches and riboregulators,^9,10^ toehold-mediated strand displacement,^11^ split T7 RNA polymerase,^8^ and CRISPR-Cas,^12^ whereas the use of ribonucleoprotein (RNP) complexes as biosensors in cell-free transcription-translation (TX-TL) systems has remained largely unexplored. Investigating their applicability in CFS could be of particular interest for novel means of detecting molecular species that operate at the posttranscriptional level, such as microRNAs (miRNAs) and viral proteases.

miRNAs are short (18–22 nucleotides) noncoding RNAs that mediate RNA interference (RNAi) through the formation of the RNA-induced silencing complex (RISC) RNP. Disease-specific miRNA signatures found in the circulation and other body fluids are promising biomarkers for noninvasive diagnostic procedures,^13–15^ highlighting an urgent need for rapid and robust miRNA detection methods that could outperform current PCR-based technologies.^16^ The development of in vitro miRNA detection through cell-free platforms has recently gained more attention thanks to the implementation of signal amplification modules that rely on CRISPR-Cas12/13,^17–19^ isothermal amplification,^20,21^ or a combination of both,^6,22,23^ allowing ultrasensitive detection.^24^ In such systems, miRNA sensing is mostly accomplished through the binding of a purified target miRNA with enzymatic effectors that elicit a measurable response. However, physiologically, miRNAs form a complex with their RNA-cleaving enzymatic partner, Argonaute-2 (Ago2), to which they stably bind.^25^ As miRNA-Ago2 complexes remain enzymatically active following isolation,^26–28^ the whole complex, instead of purified miRNAs, could be harnessed to induce an siRNA-like RNAi response coupled to cell-free protein synthesis for fast and simple signal detection. However, only a small number of studies have implemented such a sensing strategy^29,30^ and, to our knowledge, the detection capability of the RISC:*miRNA* complex in TX-TL cell-free systems has not been addressed yet.

Other synthetic circuits based on heterologous RNP complexes, such as the MS2:*ms2L* and L7Ae:*k-turn,* have been previously designed and implemented in mammalian cells as viral protease sensors,^31^ relying on the protease-dependent derepression of RNA-binding proteins (RBPs) that inhibit the expression of target RNAs at the posttranscriptional level.^32,33^ In the MS2:*ms2L* complex, the phage-derived MS2 coat protein (also called MCP) is an RBP that binds to MS2 stem-loop secondary structures (hereafter called ms2L) usually placed in the 3′ UTR of a target RNA.^34–36^ In mammalian cells, the deadenylase CNOT7 fused to MS2 downmodulates the expression of EGFP reporters carrying 8 repeats of ms2L by destabilizing the MS2-tethered RNA through deadenylation.^31,32^ The L7Ae:*k-turn* system is an RNP complex composed of an archaeal RBP, L7Ae, which binds specifically to box C/D sequences that form kink-turn (k-turn) RNA secondary structures.^37–39^ When the k-turn structure is placed in the proximity of the start codon, L7Ae represses translation in vitro and in vivo,^32,33^ likely by blocking access to the ribosome through steric hindrance. MS2-CNOT7 and L7Ae were engineered to carry a tobacco etch virus protease (TEVp) cleavage site (also called TCS) such that the conditional derepression of a reporter carrying RNA secondary structures could be obtained upon TEVp expression in cells,^31,40^ indicating their potential as virus-specific, protease-responsive biosensors. Notably, orthogonal RBP pairs developed from L7Ae:*k-turn* directed evolution^41^ have been used to build complex cell-free TX-TL circuits that use the L7Ae:*k-turn* interaction as a protein-responsive ON riboswitch,^42,43^ indicating how this RBP system could also be converted into a translational activator in addition to its traditional use as an inhibitor^33,44^ and highlighting the high versatility potential of this RBP pair in prospective translational applications. While L7ae:*k-turn* applications in CFS beyond regulation of translation have been reported,^45–49^ the functionality of MS2-CNOT7:*ms2L* RNP has not yet been investigated. Moreover, the functional characterization of additional protease-responsive control layers in MS2:*ms2L* and L7ae:*k-turn* reconstituted systems is currently lacking.

Here, we explored whether RNP complexes that have been widely used in mammalian cells to modulate RNA expression posttranscriptionally could be reconstituted in various CFSs to evaluate their potential implementation as a prospective new category of in vitro biosensors. We initially investigated the rabbit reticulocyte lysate (RRL) as a cell-free platform to design and test RISC:*miRNA* and MS2-CNOT7:*ms2L* posttranscriptional circuits, taking advantage of the lysate's characteristic presence of high miRNA abundance and the eukaryotic context allowing full CNOT7 activity, respectively. We found that both sensing platforms suffer from limitations due to topological and lysate-specific constraints, which require further optimization. Instead, the L7Ae:*k-turn* system was consistently reconstituted and thoroughly characterized using the RRL, the *E. coli* S30 lysate, and the PURE system through a stepwise optimization approach. Systematic characterization of k-turn sequences and plasmid backbones, combined with expression protocol optimization, enabled the development of a robust DNA-encoded L7Ae:*k-turn* circuit in the PURE system, bypassing the need to add L7Ae as purified protein in the reaction. As proof of principle of the feasibility to leverage the L7Ae:*k-turn* RNP complex as a protease-sensing platform, we introduced an additional layer of control exemplified by TEVp-mediated relief of TCS-carrying L7Ae inhibition and demonstrated how titrating the RBP-encoding plasmid and the TEVp amount could achieve almost complete rescue of the reporter expression.

Our results lay solid foundations for the future development of a new class of biosensors, by highlighting the RNP complexes that require further technical (RISC:*miRNA*) or mechanistic (MS2-CNOT7:*ms2L*) characterization and those that show high potential to be further implemented as stimulus-responsive biosensors (L7Ae:*k-turn*), displaying both functionally robust in vitro reconstitution and tunable protease responsiveness.

## Results

### Performance Evaluation of RNA-Based miRNA Sensors in RRL

To test the performance of miRNA sensors in a complex mixture, we leveraged the characteristics of the RRL, a mammalian translation (TL)-based cell-free expression system widely used both as a protein expression platform^50^ and as an in vitro model system for the characterization of eukaryotic translation.^51^ In addition to functional translation machinery, the RRL contains RISC components and endogenous miRNAs, persisting even upon micrococcal nuclease treatment.^52^ Each reporter consists of a capped- and polyadenylated RNA produced by in vitro transcription (IVT) carrying the firefly luciferase (FLuc) coding sequence (CDS) and 4 tandem repetitions (4×) of specific miRNA target sites (TSs) in the 3′ UTR, responding to endogenous miRNAs expressed at high (miR-451a), medium (let-7a), and low (miR-221) levels in the RRL.^52^ Importantly, TS was designed to be fully complementary to their cognate miRNA to activate an siRNA-like RNAi response in RRL, which leads to the cleavage of the target RNA followed by its degradation.^52^ As a result, FLuc translation levels (measured by luminescence output) of each miRNA sensor are expected to negatively correlate with the concentration of the cognate miRNA present in the RRL (Figure 1A). To sense endogenous miRNAs in the RRL, we followed the protocol published by Ricci and colleagues (2010)^52^ that untangles the processes of translation and miRNA downmodulation by incubating the sensor RNA within the lysate at low temperature (10 °C), allowing the endogenous RISC:*miRNA* complex to anneal to its target while translation is halted (Figure S1A). As a “baseline” condition, consisting of the maximum translation potential of each FLuc RNA reporter, we added specific synthetic anti-miRNAs (α-miRNAs) that neutralize the activity of the miRNA of interest before adding the RNA target carrying the miRNA TS (Figures 1B and S1A). The translation of reporter RNAs carrying miR-451a or let-7a TS was rescued only when the cognate α-miRNA was added to the RRL lysate (Figure 1B,C), indicating that each reporter RNA is acting as a sensor of the cognate miRNA in a specific manner. However, the translation levels of the reporter RNA carrying miR-221 TS were unchanged across all conditions, suggesting two possible explanations. On one hand, miR-221 concentration might fall below the limit of detection of the miRNA sensor, consistent with the absence of rescue of the cognate α-miRNA. On the other hand, miR-221 might downmodulate the reporter RNA, with the cognate α-miRNA failing to restore its basal levels. A more precise quantification of each miRNA in RRL by absolute qPCR (Figure 1D) confirmed the differential concentration of the 3 miRNAs, although we found that miR-221 was more concentrated than previously shown.^52^ To improve signal sensitivity and dynamic range, we replaced the CDS of FLuc with that of NanoLuc (NLuc), an engineered luciferase with a specific activity ∼150-fold greater than that of FLuc.^53^ The results obtained with NLuc RNA reporters (Figure 1E) were similar to those of FLuc (Figure 1B), although the luminescence intensity was ∼2 orders of magnitude higher in the case of NLuc reporters. Since commercial RRL is optimized for translation efficiency by the supplementation of translation-enhancing factors, we hypothesized that miRNA TS in the 3’ UTR of reporter RNAs might underestimate miRNA-mediated activity, with highly efficient translation initiation at the 5′ UTR “competing” with miRNA-mediated RNA degradation in the 3′ UTR. To untangle these two possibly competing phenomena, we produced NLuc-carrying reporter RNAs with miRNA TS at the 5′ UTR instead of the 3′ UTR. The dynamic range of miRNA-mediated inhibition of both miR-451a and let-7a reporter RNA significantly improved, with the 5′-TS reporter RNAs performing significantly better than 3′-TS reporters in sensing the 3 different miRNA concentrations (Figure S1B) (3′-TS: not significant; 5′-TS: *p* < 0.05; correlation analysis). However, no change was again observed for the miR-221 reporter between the control (H_2_O) and α-miRNA condition (Figure 1F). Due to the lack of straightforward ways to (i) validate the “sponging” activity of miR-221 α -miRNA and (ii) test the impact of translation-inhibiting secondary structures that form upon the insertion of each miRNA TS, the ability of miR-221 sensor RNA to detect its cognate miRNA in RRL remains to be further investigated. Moreover, other variables, unrelated to RRL, could have impacted RNA translation, such as the presence of unknown miRNA homologues and the occurrence of unspecific RNA degradation-related issues often difficult to control. For these reasons, we concluded that miRNA biosensors based on IVT RNAs expressed in CFS need further thorough optimization to control the variables listed above, currently limiting their potential as an RNA-based platform to interrogate miRNA levels with acceptable sensitivity and confidence.

**Figure 1:**
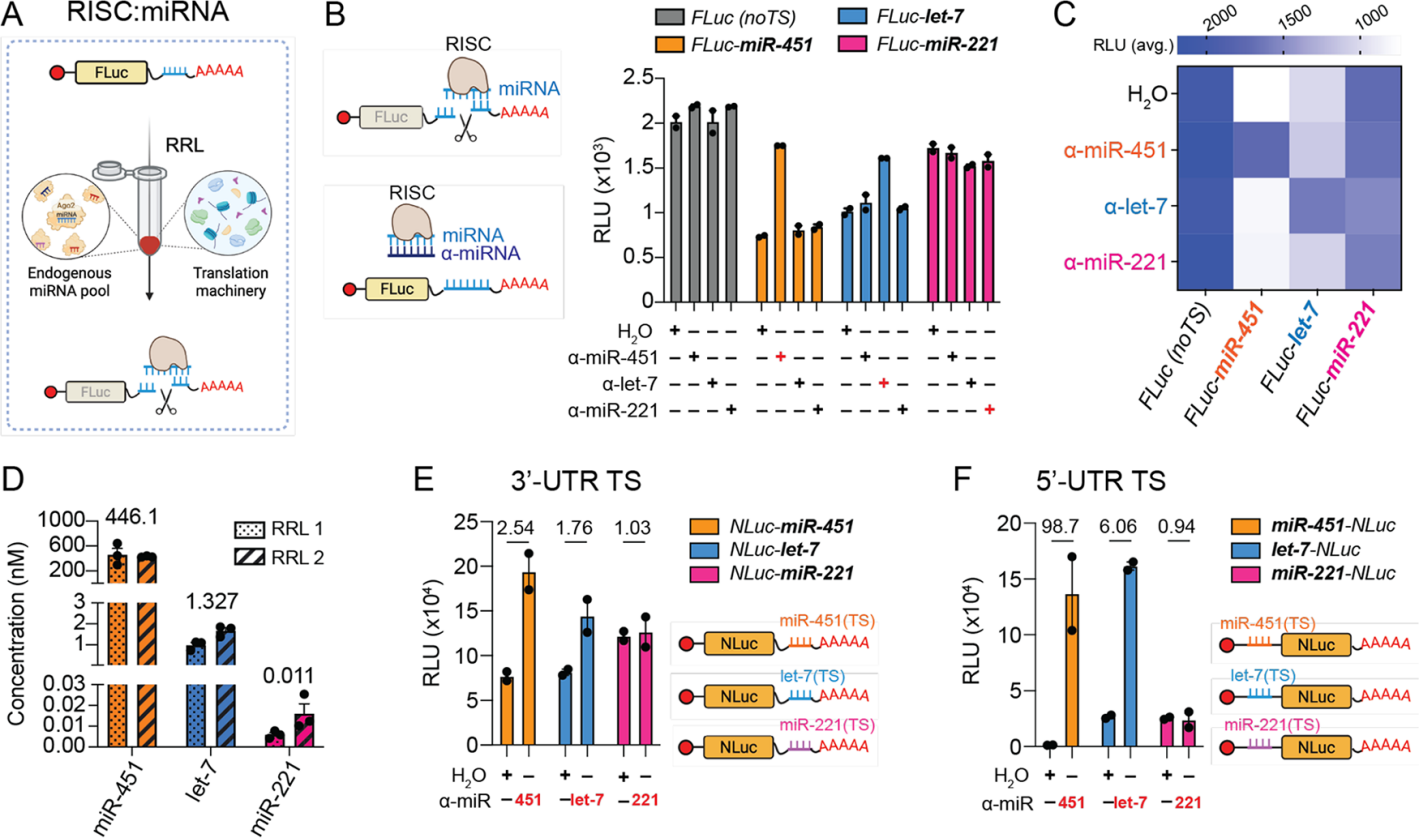
RNA-based microRNA sensors in RRL. (A) RNAs encoding the firefly luciferase (FLuc) reporter, including miRNA target sites (TSs) in their 3′ UTR, are produced by in vitro transcription (IVT), capped and polyadenylated, and subsequently introduced in RRL. If the target miRNA is present, the RISC:*miRNA* complex anneals to FLuc and initiates the cleavage of the reporter under translation-repressive conditions, resulting in lower FLuc expression as compared to RNAs lacking the TS. (B) Translation levels of IVT FLuc reporter RNAs carrying either no miRNA target sites (“noTS”) or TS responding to miR-451a (orange), let-7a (blue), or miR-221 (magenta). The degree of reporter translation rescue, indicating miRNA specificity to the TS, was evaluated by preincubating synthetic anti-miRNAs (a-miRNAs) or water in the RRL prior to reporter addition. Cognate α-miRNA:miRNA reporter pairs are indicated in red. (C) Heatmap summarizing average luminescence intensities measured in (B) with α-miRNA (or H2O) (*y* axis) vs miRNA reporters (*x* axis). Cognate α-miRNA:miRNA TS are color-paired. (D) miRNA quantification (nM) by qPCR in two different RRL aliquots (RRL1 and RRL2). Numbers above bars indicate the average concentration (nM) of the respective miRNA. (E, F) Average luminescence of nanoluciferase (NLuc) reporters carrying miRNA TS at their 3′ UTR (E) or at their 5′ UTR (F) with the α-miRNA vs H20 condition fold change indicated above the respective bars. The a-miRNA used for NLuc rescue (cognate α-miRNA) is indicated below each graph in red. Values are indicated as mean ± s.e.m (B, D–F). RNA regulatory elements (miRNA TS) are indicated in bold. RLU: relative luminescence unit.

### Independence from PolyA and MS2-Tethering Limits the Use of MS2-CNOT7:*ms2L* as In Vitro Biosensor

For its attractive potential as an in vitro biosensor, we tested if the MS2-CNOT7-based RNP system could be recapitulated in the mammalian RRL cell-free system (Figure 2A). Starting from capped and polyadenylated RNAs encoding an *FLuc-ms2L* reporter and an MS2-CNOT7 repressor, we first developed a TL protocol that consisted of the preincubation of the MS2-CNOT7 RNA in the CFS to allow its translation, followed by the addition of the *FLuc-ms2L* reporter to the MS2-CNOT7-containing RRL (see Materials and Methods for further details). Since the reporter signal output is heavily dependent on the resources that are available in the cell-free extract,^54,55^ we introduced a control condition where the RNA encoding a binding-inefficient version of MS2 (MS2*)^56^ was fused with CNOT7 and coexpressed with the *FLuc-ms2L* reporter (Figure 2B) as indicated earlier. This allowed us to account for the same rate of translation resource consumption between the control and test conditions (each composed of a total RNA pool of the same amount and length), minimizing signal artifacts due to differential resource availability that occur when RNAs of different lengths or concentrations are translated.^54,57^ Finally, we introduced a condition in which the control MS2*-CNOT7 or the wild-type MS2-CNOT7 was mixed with *FLuc-ms2L* lacking the polyA tail to test if the polyA presence was necessary for CNOT7-mediated repression (Figure 2B). In accordance with previous studies that reported polyA-independent translation in nuclease-treated RRL,^58,59^ we confirmed that *FLuc-ms2L* RNAs lacking the polyA are efficiently translated in the RRL, even at higher levels than their polyadenylated counterparts (Figures 2C and S2). Interestingly, no significant difference in *FLuc-ms2L* translation was observed between the negative control (MS2*-CNOT7) and the test (MS2-CNOT7) conditions (*p* > 0.05; one-tailed paired *t* test), regardless of polyA addition to the reporter RNA (Figure 2C). This phenomenon might be due to the presence of functional CNOT7 on both control and test RBPs, likely inhibiting RNA translation regardless of MS2-mediated tethering in the RRL context. Indeed, *FLuc-ms2L* reporters coexpressed with MS2-only RBPs (either wild-type or binding-inefficient) displayed on average ∼4× more signal than their CNOT7 fusion counterparts (Figure 2D). Moreover, wild-type MS2-CNOT7 repression activity was independent of the presence of *ms2L* secondary structures on FLuc RNA reporters (Figure 2E).

**Figure 2:**
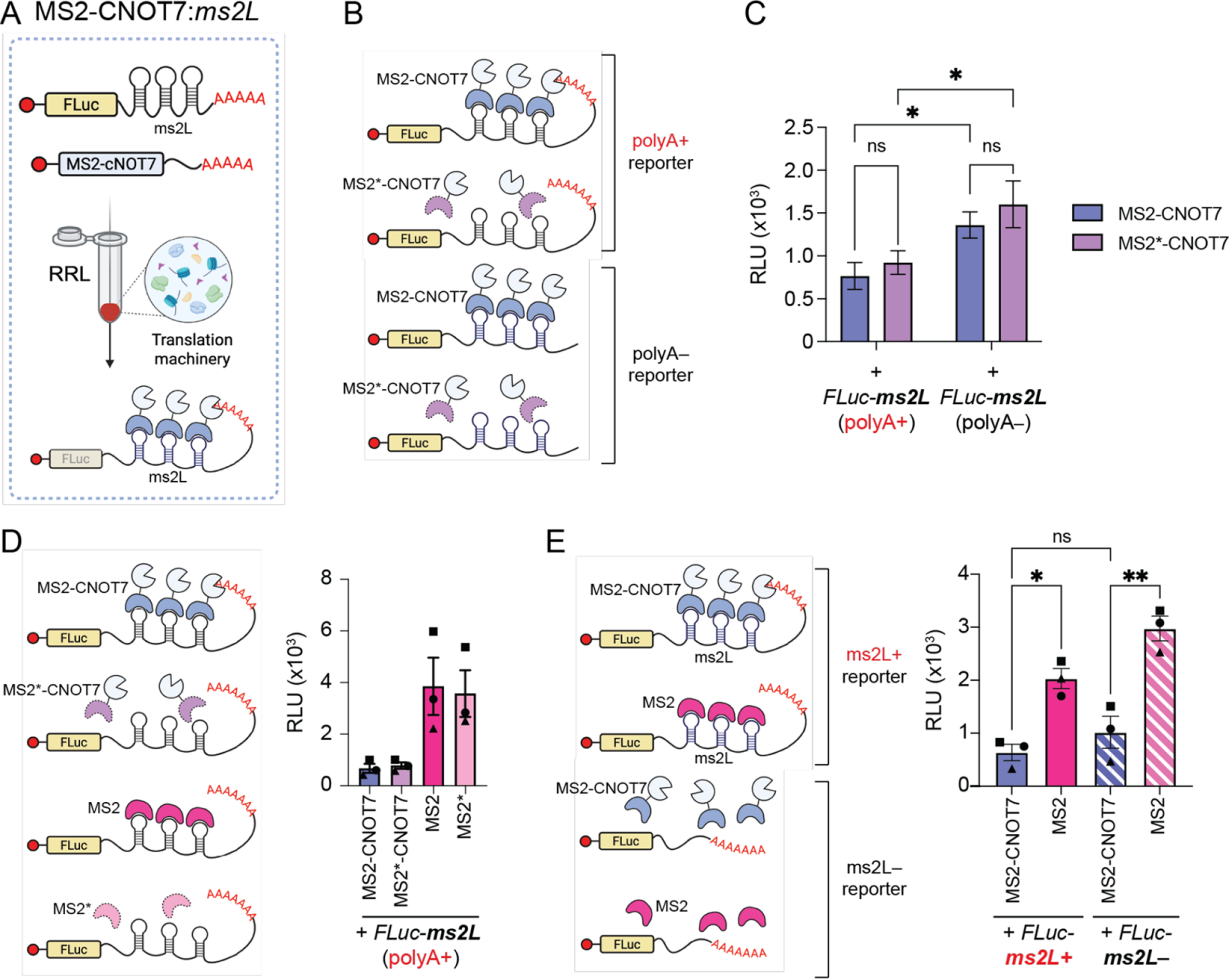
Characterization of the MS2-CNOT7:*ms2L* system in RRL. (A) Schematic of MS2-CNOT7 translational repression on MS2 loops (ms2L)-carrying FLuc reporter in RRL. Capped and polyadenylated MS2-CNOT7 RNA is preincubated in the RRL to allow translation of the repressor. Once introduced in the RRL containing the MS2-CNOT7 protein, the translation of capped and polyadenylated *FLuc-ms2L* RNA reporter is inhibited by CNOT7-mediated deadenylation of the FLuc reporter RNA through MS2:ms2L tethering. (B) Schematic of reporter-repressor pairs tested for polyA-mediated repression. Capped polyA+ or polyA-*FLuc-ms2L* RNA reporters were incubated in RRL containing pretranslated wild-type (MS2-CNOT7) or mutated (MS2*-CNOT7, lacking ms2L-binding capacity) chimeric RBP. (C) Evaluation of wild-type or mutated MS2-CNOT7-mediated repression of polyA+ (left) or polyA- (right) FLuc-ms2L reporter RNA. Values indicate mean luminescence ± s.e.m. (*n* = 6). Statistical significance was evaluated with two-way ANOVA followed by Fisher’s LSD test. (D) Evaluation of MS2-mediated repressing activity on capped and polyadenylated *FLuc-ms2L* RNA reporters with the RNA:RBP pairs indicated on the left. Values indicate mean luminescence ± s.e.m (*n* = 3). (E) Evaluation of the impact of MS2-mediated tethering of CNOT7 on FLuc reporters with (*FLuc-ms2L+*) or without (*FLuc-ms2L-*) ms2L. RNA:RBP pairs are indicated on the left. Values indicate mean luminescence ± s.e.m (*n* = 3). Statistical significance was evaluated with one-way ANOVA followed by Fisher’s LSD test. **p < 0.01; *p < 0.05; ns = not significant. RNA regulatory elements (ms2L) are indicated in bold. RLU = Relative Luminescence Unit.

To test whether CNOT7 was exerting a concentration-dependent unspecific effect by eliciting general RNA degradation, we titrated the amount of repressor-encoded RNA (70, 140, or 280 fmol) in translation reactions where the amount of *FLuc-ms2L* reporter RNA was kept constant (70 fmol). In parallel, we added a condition where only the *FLuc-ms2L* reporter was translated (“H_2_O”) to have an indication of the “translation capacity” of different lysate batches (Figure S3A). By increasing the concentration of the repressor, we observed that the average reporter expression increased proportionally in both the MS2-CNOT7 and MS2*-CNOT7 conditions when accounting for lysate translation capacity (Figure S3B). However, we observed the opposite effect on translation output than the one expected when a higher amount of RNA is added to the lysate, which usually results in a lower reporter protein synthesis due to increased resource consumption. Interestingly, this phenomenon might indicate counteracting effects on translation by CNOT7 activity within the lysate. Since deadenylated RNAs are translated more efficiently in the RRL (Figures 2C and S2), we hypothesized that, while the deadenylating activity of CNOT7 enhances the translation of *FLuc-ms2L* RNA by removing the polyA, an unknown, deadenylase-independent mechanism imparts a translation-repressive activity on the reporter. In support of this hypothesis, the repression fold change does not linearly correlate with the amount of repressor RNA added (Figure S3C). Overall, these results suggest that the dual activity of CNOT7, composed of a well-characterized deadenylating component^60^ and another unknown polyA-independent inhibiting function,^36^ leads to counteracting effects in modulating the translation of RNAs in the RRL, due to the RRL-specific characteristic of enhanced protein synthesis of deadenylated RNAs.

Since a clear loss of RNA repression linearly correlating with a gradual loss of CNOT7 activity upon tethering disruption would be necessary for building a protease-responsive RBP cell-free biosensor, we considered MS2-CNOT7 not suitable for biosensor development in the RRL cell-free context without further structural and functional studies of CNOT7 mechanism of action that go beyond the scope of this work.

### RNA-Encoded L7Ae Significantly Inhibits the Expression of Reporter RNAs Carrying k-Turn Structures in RRL

Previous experiments conducted in the PURE CFS had shown that the addition of purified L7Ae protein effectively represses the reporter RNAs carrying k-turn structures near the ATG.^33^ Therefore, we aimed to test whether RNA-encoded L7Ae constructs were also effectively blocking the translation of *K-turn-FLuc* RNAs in the RRL (Figure 3A). To this end, we designed and produced RNA-encoded (i) repressors—L7Ae or L7Ae­(TCS)—and (ii) *K-turn-FLuc* reporters, either carrying a functional (*K-turn-FLuc*) or nonfunctional (K-turn*-FLuc) box C/D sequence (Figure 3B). Using the same preincubation protocol described earlier to initiate L7Ae protein synthesis, followed by reporter RNA addition, we found that L7Ae represses *K-turn-FLuc* reporters in a small but significant manner (*p* < 0.01; one-tailed paired *t* test) (Figure 3C). However, L7Ae­(TCS) failed to significantly repress the *K-turn-FLuc* reporter (*p* > 0.05, one-tailed paired *t* test) (Figure 3C). Given the modest effect of wild-type L7Ae on *K-turn-FLuc* RNA, it is likely that the effect exerted by L7Ae­(TCS), previously described as less efficient than its wild-type counterpart in repressing reporter translation,^31,61,62^ would be too small to be visible in the RRL assay. To circumvent potential issues that might be difficult to control when working with RNA, we switched from an RNA-based translation-only CFS (RRL) to DNA-based TX-TL CFSs (such as the *E. coli* S30 lysate and the PURE) for further characterization and optimization of the promising L7Ae:*K-turn* repression system.

**Figure 3:**
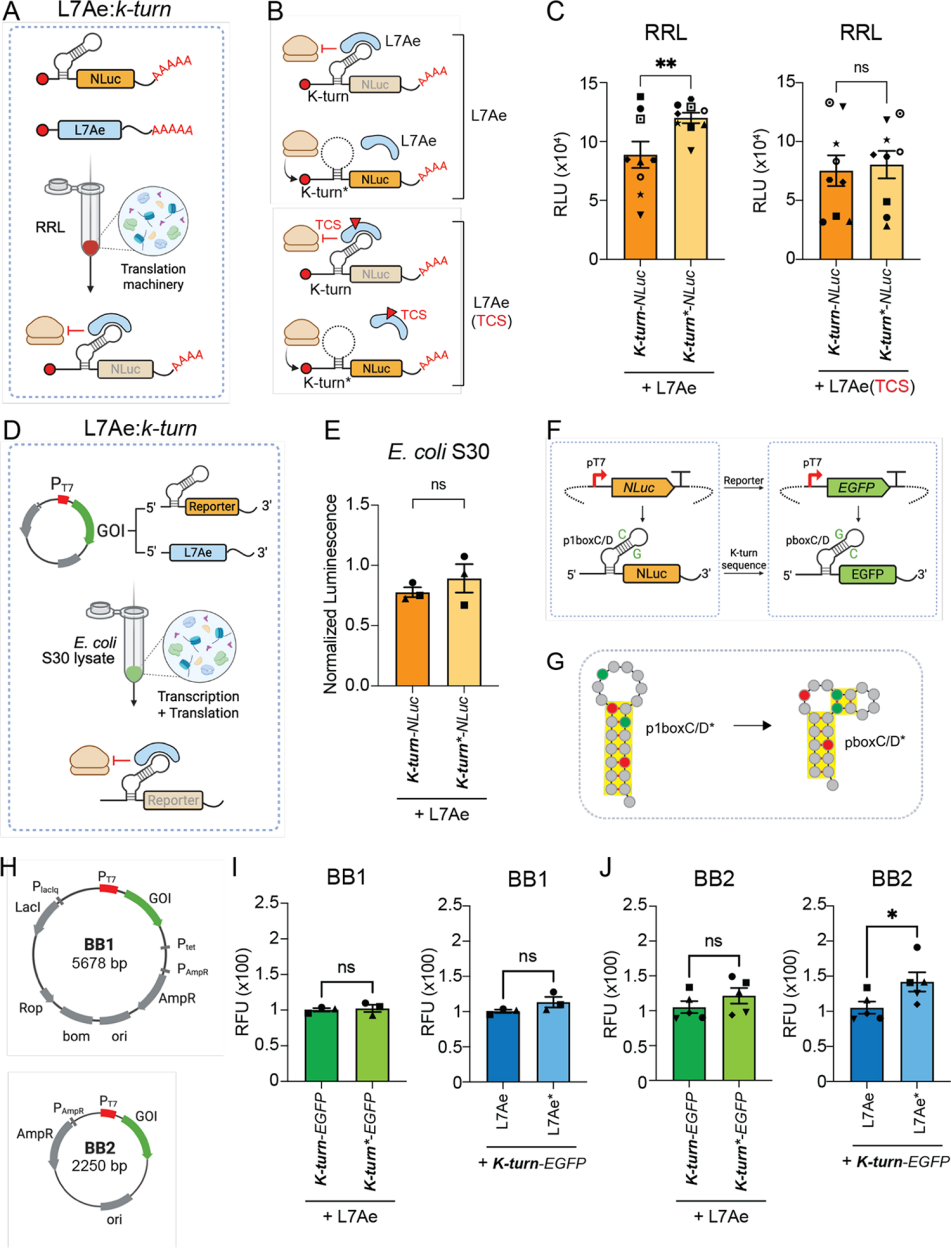
Characterization of L7Ae:*k-turn* system in the RRL and *E. coli* S30 lysate. (A) Schema c of L7Ae-mediated translational repression of NLuc reporter carrying the L7Ae binding motif of k-turn immediately downstream of the NLuc start codon. Capped and polyadenylated L7Ae RNA is preincubated in the RRL to allow translation of the repressor. When *K-turn-NLuc* RNA is added to the RRL, its translation is inhibited by L7Ae binding to the k-turn, due to halting ribosomal recruitment and translation initiation. (B) Schematic of reporter-repressor pairs tested for repression efficiency. Capped and polyadenylated NLuc RNA reporters carrying a wild-type (*K-turn-NLuc*) or a mutated (K-turn*-NLuc) secondary structure were incubated in RRL containing pretranslated wild-type L7Ae or L7Ae carrying a TEV cleavage site (TCS) consensus sequence for layered protease regulation. (C) Evaluation of the repression efficiency of L7Ae (left) or L7Ae (TCS) (right) on NLuc reporter RNAs carrying the k-turn or k-turn* secondary structure. Values indicate mean luminescence ± s.e.m (*n* = 9). Statistical significance was evaluated with a one-tailed paired *t* test. (D) Schematic of L7Ae translational repression on k-turn-carrying reporters in *E. coli* S30 TX-TL lysate. The coding sequences of both the repressor and the reporter were placed under the control of the T7 promoter (PT7). (E) Mean luminescence values of *K-turn-NLuc* and K-turn*-NLuc reporters upon TX-TL expression. Luminescence values of reporter-repressor pairs were normalized to those of the respective reporter-only conditions due to differences in their translation levels (see Figure S4B). Values are indicated as mean ± s.e.m (*n* = 3). Statistical significance was evaluated with a one-tailed paired *t* test following log-transformation of reporter-normalized data. (F) Optimization of the reporter and k-turn primary sequence. NLuc CDS was substituted with EGFP CDS to minimize sources of contamination, such as the luciferase substrate, and simplify reporter detection. (G) The p1boxC/D* sequence used in k-turn* carrying the reporter was changed into the pboxC/D* sequence to reduce the chance of low translation efficiency due to the formation of strong secondary structures (see Figure S5 for further details). (H) Schematic of plasmid backbones containing the gene of interest (GOI) under the control of the PT7 used in this study to analyze the impact of plasmid complexity on the K-turn-EGFP repression by L7Ae (see Figure S7 and File S1 for further details). (I, J) Mean fluorescence of EGFP reporters (±s.e.m) upon L7Ae repression, indicating the impact of BB1 (I) and BB2 (J) plasmid backbones. Le: EGFP reporters carrying k-turn or k-turn* structures incubated with L7Ae. Right: *K-turn-EGFP* reporters incubated with wild-type L7Ae or mutated L7Ae (L7Ae*). Statistical significance was computed with a one-tailed paired *t* test (I: *n* = 3; J: *n* = 4). **= *p* < 0.01; * = *p* < 0.05; ns = not significant. RNA regulatory elements (k-turn, k-turn*) are indicated in bold. RFU = relative fluorescent unit.

### Different Box C/D Sequences on the Reporter RNA Lead to Unbalanced Reporter Translation

We first introduced plasmid-encoded L7Ae and *K-turn-NLuc* reporters in the S30 *E. coli* lysate in the absence of T7 RNA polymerase, relying only on the previously observed background level of transcription of the T7 promoter^63^ (Figure 3D). This allowed us to (i) control burden-related effects resulting from high gene expression levels and (ii) analyze the contribution of the plasmid backbone to reporter expression and inhibition efficiency. Interestingly, the expression level of the wild-type *K-turn-NLuc* reporter was ∼6× higher than the mutated K-turn*-NLuc reporter (in the absence of L7Ae) (Figure S4B), while the same constructs expressed in the RRL in the form of RNA displayed similar levels (Figure S4A). When plasmid-encoded L7Ae was introduced in the S30 *E. coli* lysate following the preincubation protocol, no significant L7Ae-mediated repression was observed (*p* > 0.05, one-tailed paired *t* test) (Figure 3E). However, although the signal was normalized to the respective reporter-only condition, we considered the differences observed in the basal expression levels of the wild-type and mutated reporter as too high to conduct a reliable assessment of the degree of inhibition. We hypothesized that the mutated k-turn construct we used could assume strong unwanted secondary structures that could severely reduce its translation potential, as suggested by in silico RNA folding predictions (Figure S5). For this reason, we replaced the mutated “p1boxC/D” (p1boxC/D*) sequence (Figure S5B) with the mutated “pboxC/D” (pboxC/D*) (Figure S5D) variant (differing only in the position of a C-G pair in the k-turn structure)^33^ and switched from the NLuc CDS to the EGFP CDS to eliminate the need of luciferase substrate addition (Figure 3F,G), which can be a source of potential contamination. These changes resulted in the restoration of the K-turn*-EGFP reporter expression levels to those of its wild-type counterpart (Figure S6A), indicating that even small changes in the k-turn sequence (wild-type or mutated) could have a profound impact on the expression levels of the reporter. For this reason, we decided to proceed with EGFP reporters carrying the pboxC/D k-turn secondary structure for further optimization of this RNP system.

### Plasmid-Based Expression of the L7Ae:*K-turn* RNP System Shows Backbone-Dependent Repression Efficiency in Both the *E. coli* S30 Lysate and the PURE

To test if the plasmid backbone affects the degree of L7Ae-mediated repression, we inserted the EGFP reporter (carrying wild-type or mutated k-turn structures), the wild-type L7Ae, and a binding-inefficient version of L7Ae (called L7Ae*)^33^ into two different backbones, BB1 and BB2 (Figure 3H) (File S1). BB1 (5678 bp) is highly complex, containing several genetic elements that can be expressed in the *E. coli* S30 lysate by endogenous factors, as well as a T7-repressing lacO sequence responsive to the LacI gene encoded in the same backbone, and results in T7-transcribed RNAs with a 147-bp 3′ UTR (Figure S7A). BB2 (2250 bp) contains only the necessary elements for plasmid replication and selection for cloning purposes, does not contain any lacO-LacI sequences to limit the leakiness of the T7 promoter, and results in T7-transcribed RNAs with a 195-bp 3′ UTR (Figure S7B). Interestingly, when the L7Ae:*K-turn* RNP system was encoded in BB1, L7Ae-mediated repression was smaller than the inhibition observed with the BB2-encoded circuit (Figure 3I,J). However, it is well-known that the *E. coli* S30 extract is highly complex, with a proteome composition that remains difficult to identify or control.^64^ For this reason, we decided to further optimize the cell-free reconstitution of L7Ae:*k-turn* RNP complex in a more controlled and standardized cell-free system, such as the PURE^65–67^ (Figure 4A). We then tested the degree of L7Ae repression on *K-turn-EGFP* reporters encoded in the same BB1 or BB2 plasmid backbones used in the *E. coli* S30 lysate (Figure 4B). L7Ae inhibitory activity on the *K-turn-EGFP* reporter was stronger when constructs were encoded in the BB2 plasmid (Figure 4C). However, differences in the inhibition of *K-turn-EGFP* reporters by L7Ae wild-type and mutated variants were more pronounced when the constructs were encoded in the BB1 plasmid (Figure 4D). Interestingly, the PURE system is characterized by higher absolute expression levels of BB1-encoded constructs than BB2-encoded ones (Figure S6B), in contrast to what was observed for *E. coli* S30 lysate (Figure S6A). To better understand this phenomenon, we performed a series of titration experiments in which the amount of BB1- or BB2-encoded *K-turn-EGFP* reporter plasmid was held constant (25 fmol), while increasing amounts of BB1- or BB2-encoded L7Ae or L7Ae* (negative control) were added (Figures 4E and S8). Notably, increasing the amount of repressor plasmids led to a reduction of absolute EGFP expression levels in all conditions, likely resulting from resource competition.^68–70^ Interestingly, the repression rate of L7Ae remained constant when the repressor was encoded in BB1, either with BB1- (Figure S8A,E) or BB2-encoded (Figure S8C,G) EGFP reporter. In contrast, the L7Ae repression rate increased when the repressor was encoded in BB2 (Figure S8B,D), with a significant correlation between the amount of L7Ae plasmid introduced and repression achieved, although it never reached the levels observed when the repressor was encoded in the BB1 backbone (*p* < 0.05, simple linear regression analysis) (Figure S8F,H). The lower expression levels of BB2-encoded EGFP reporters observed in PURE (Figure S6B) suggest that also BB2-encoded L7A/L7Ae* is produced at lower levels than BB1-encoded repressors, failing to exert a significant repression unless higher amounts of repressor plasmid are added. Moreover, it is possible that the mutations introduced in the L7Ae* do not completely inactivate the binding capacity of the RBP, leading to incomplete rescue of the EGFP signal.

**Figure 4:**
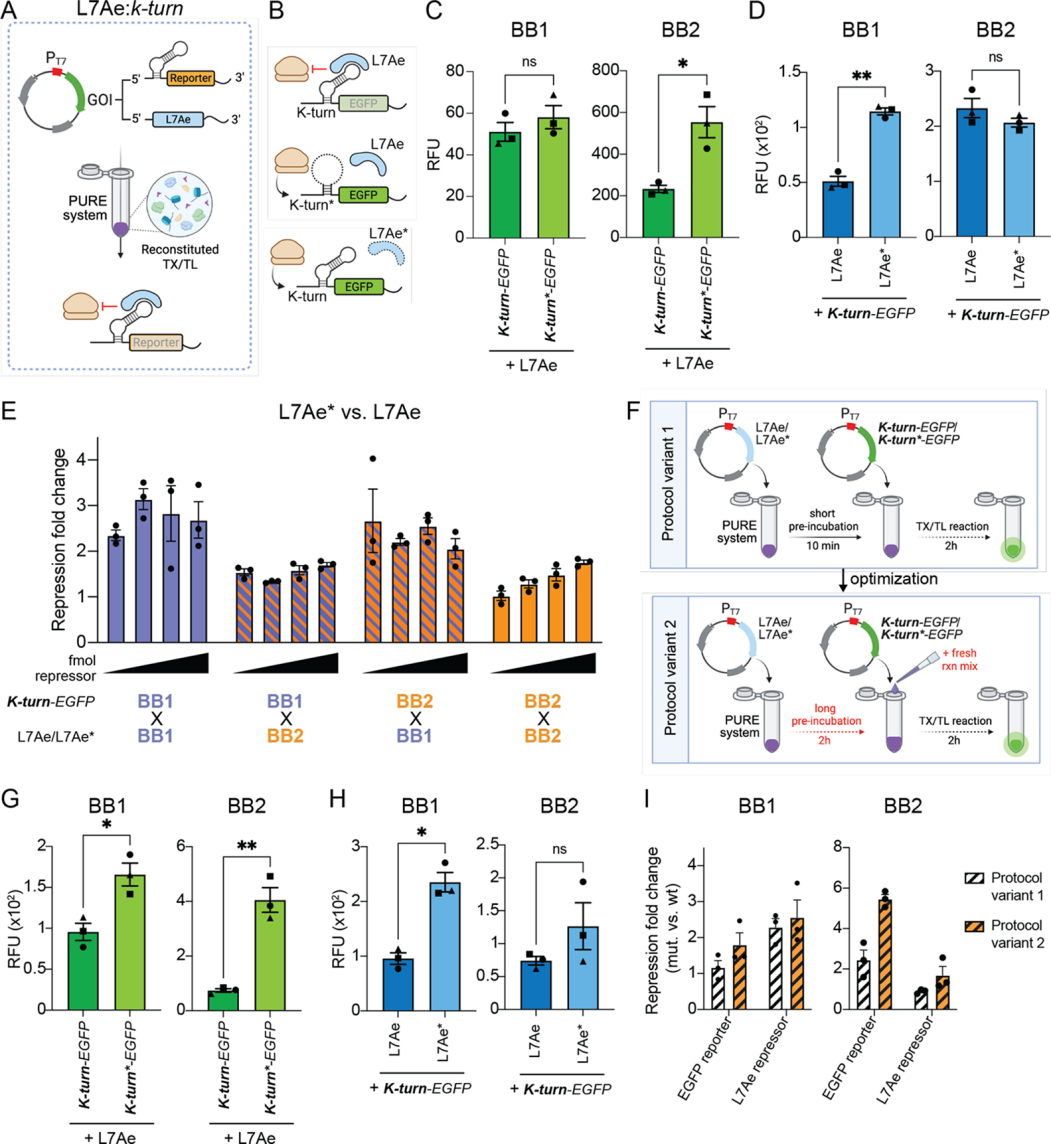
Optimized protocol in the PURE expression system for L7Ae:*k-turn* performance evaluation. (A) Schematic of L7Ae translational repression on k-turn-carrying reporters in the PURE expression system. Plasmid-encoded repressors and reporters under the control of the T7 promoter (PT7) are introduced in the PURE system, where the T7 RNA polymerase and purified *E. coli* proteins initiate TX and TL. (B) Schematic of reporter-repressor pairs tested for repression efficiency in PURE. (C, D) Mean fluorescence of EGFP reporters upon L7Ae repression encoded in BB1 (le) or BB2 (right) backbones. In C, EGFP reporters carrying k-turn or k-turn* structures were incubated with L7Ae; in D, *K-turn-EGFP* reporters were incubated with wild-type L7Ae or mutated L7Ae (L7Ae*). Statistical significance was computed with a one-tailed paired *t* test (*n* = 3). (E) Repression fold change of K-turn-EGFP reporter upon L7Ae* vs L7Ae repression encoded in BB1 or BB2 backbones at varying repressor plasmid concentrations (fmol, *x* axis). Raw EGFP expression levels are displayed in Figure S8. Values are indicated as mean ± s.e.m. (F) Schematics of the TX-TL protocol before (protocol variant 1, upper panel) and after optimization (protocol variant 2, lower panel) to enhance L7Ae-mediated repression in PURE. (G, H) Mean fluorescence of EGFP reporters upon L7Ae repression encoded in BB1 (le) or BB2 (right) backbones obtained by following protocol variant 2. Statistical significance was computed with a one-tailed paired *t* test (*n* = 3). (I) Repression fold change obtained with each protocol variant for plasmids with BB1 (left) or BB2 (right) backbones. Fold change values were obtained by dividing the EGFP expression levels in the mutated vs wild-type conditions in C–D (protocol variant 1) and G–H (protocol variant 2). **p < 0.01; *p < 0.05; ns = not significant. RNA regulatory elements (k-turn, k-turn*) are indicated in bold. RFU = Relative Fluorescent Unit.

### Plasmid-Based Expression of the L7Ae:*k-turn* RNP System Results in Robust Repression upon Protocol Optimization

We hypothesized that the lower expression levels linked to the BB2 backbone and partial L7Ae* loss of function might explain the positive correlation between repression fold change and BB2-encoded repressor amount (Figures 4E and S8F,H). However, BB2-encoded *K-turn-EGFP* reporters show better repression compared with BB1-encoded ones by the wild-type L7Ae RBP (Figure 4C), indicating an apparent discrepancy in our hypothesis. We noticed that the absolute expression levels of BB1-encoded EGFP reporters were considerably lower than BB2-encoded ones, even under the K-turn*-EGFP or L7Ae* negative control conditions (Figure S9A,B). Therefore, we thought that the discrepancy observed above was due to a suboptimal timing in our expression protocol, leading to possibly biased results from an incomplete TX-TL. To test this, we adjusted the protocol previously used by (i) extending the preincubation of the repressor (L7Ae or L7Ae*) plasmid from 10 to 120 min to increase the inhibitor protein synthesis rate and (ii) adding, together with the *K-turn-EGFP* or K-turn*-EGFP plasmids, fresh PURE reaction mix to restore transcription and translation factors that may have been depleted during the repressor preincubation step (Figure 4F and Materials and Methods). Following protocol optimization, we observed that absolute reporter expression values under negative control conditions were higher for BB1-encoded constructs (Figure S9C) but did not change significantly for BB2-encoded constructs (Figure S9D). Such an optimized expression, however, improved the repression exerted by L7Ae on *K-turn-EGFP* in all conditions (Figure 4G,H), with an overall improvement of the repression fold change compared to K-turn*-EGFP or L7Ae* nonrepressing controls (Figure 4I). Altogether, these results indicate that the L7Ae repression rate relies on the amount of protein produced, requiring a thorough expression protocol timing adjustment to allow the production of sufficient repressor by TX-TL. Nevertheless, further experiments are needed to study the impact that different backbones exert on expression outputs, focusing on dissecting the contribution of single genetic elements and plasmid length, which might have both activating and repressing effects on gene expression.

### Protease-Responsive L7Ae:*K-turn* RNP Circuit in PURE Successfully Recapitulates TEVp-Mediated Rescue

Since we could not confidently measure the L7Ae/L7A* protein output expressed alone with the two backbones, as we did for the EGFP reporters, we decided to further develop a TEVp-responsive L7Ae RBP by using the K-turn*-EGFP control condition instead of the L7Ae* condition, focusing in particular on BB2-encoded circuits which displayed the highest (∼5.5×) repression fold change (Figure 4G). Differently to what observed for the RRL (Figure 3C), with our improved expression protocol we successfully obtained a significant repression (*p* < 0.05; one-tailed paired *t* test) by both wild-type L7Ae and the engineered variant L7Ae­(TCS) on *K-turn-EGFP* reporter in the PURE system (Figure 5A), confirming the robustness of the L7Ae:*k-turn* reconstitution in PURE. To proceed with our development of a protease-responsive L7Ae:*k-turn* RNP system in vitro (Figure 5B), we first tested whether we could implement a TEVp addition step to our optimized translation protocol, as optimal TEVp activity may benefit of lower working temperatures and protease buffer addition. Neither TEV buffer addition (Figure S10A,B) nor a lower (30 °C) working temperature (Figure S10C) considerably impacted L7Ae­(TCS)-mediated inhibition of *K-turn-EGFP* reporter. Therefore, we further optimized our translation protocol such that all translation reactions were carried at 30 °C and added a recombinant TEVp addition step after 1h of L7Ae­(TCS) translation (Figure 5C). When we used a molar ratio of 1:3 reporter:repressor (EGFP:R), 5 U of TEVp were sufficient to obtain 32% of signal rescue of the *K-turn-EGFP* reporter (Figure 5D), with a rescue efficiency that increased to 44% when 10 U of TEVp were added (Figure 5E). Finally, by increasing the EGFP:R molar ratio from 1:3 to 1:6 (using 10 U of TEVp) we managed to further improve the TEV rescue efficiency, reaching almost complete rescue (95%) with a limit of detection (LOD) of 2.83 U of TEVp (Figure 5F). By doubling the amount of repressor added to each reaction in switching from 1:3 to 1:6 EGFP:R molar ratio, the EGFP absolute signal decreased in all conditions (Figure 5F), probably due to a higher rate of resource consumption in the repressor preincubation phase, as discussed above. Nevertheless, L7Ae­(TCS)-mediated repression of the *K-turn-EGFP* reporter in the absence of TEVp was maintained between the 1:3 and 1:6 conditions, suggesting that in the 1:3 EGFP:R condition the repressor concentration was already saturating the k-turn sites on the reporter. However, switching to the 1:6 EGFP:R molar ratio greatly improved TEVp-mediated rescue of the EGFP signal compared to the 1:3 condition (Figures 5G and S10D). Since the TEVp amount was kept constant between the two conditions (10 U) these results suggest that the high level of rescue observed in the 1:6 condition likely emerges from indirect effects due to the differential resource consumption of the TX and TL components within the PURE extract. In fact, the 1:6 condition may use more TX-TL resources than the 1:3 condition, making rescue effects easier to detect because fewer resources are left for EGFP reporter transcription and translation. Nevertheless, further experiments will be required to thoroughly evaluate the significance of differential resource consumption on L7Ae-mediated inhibition and rescue.

**Figure 5:**
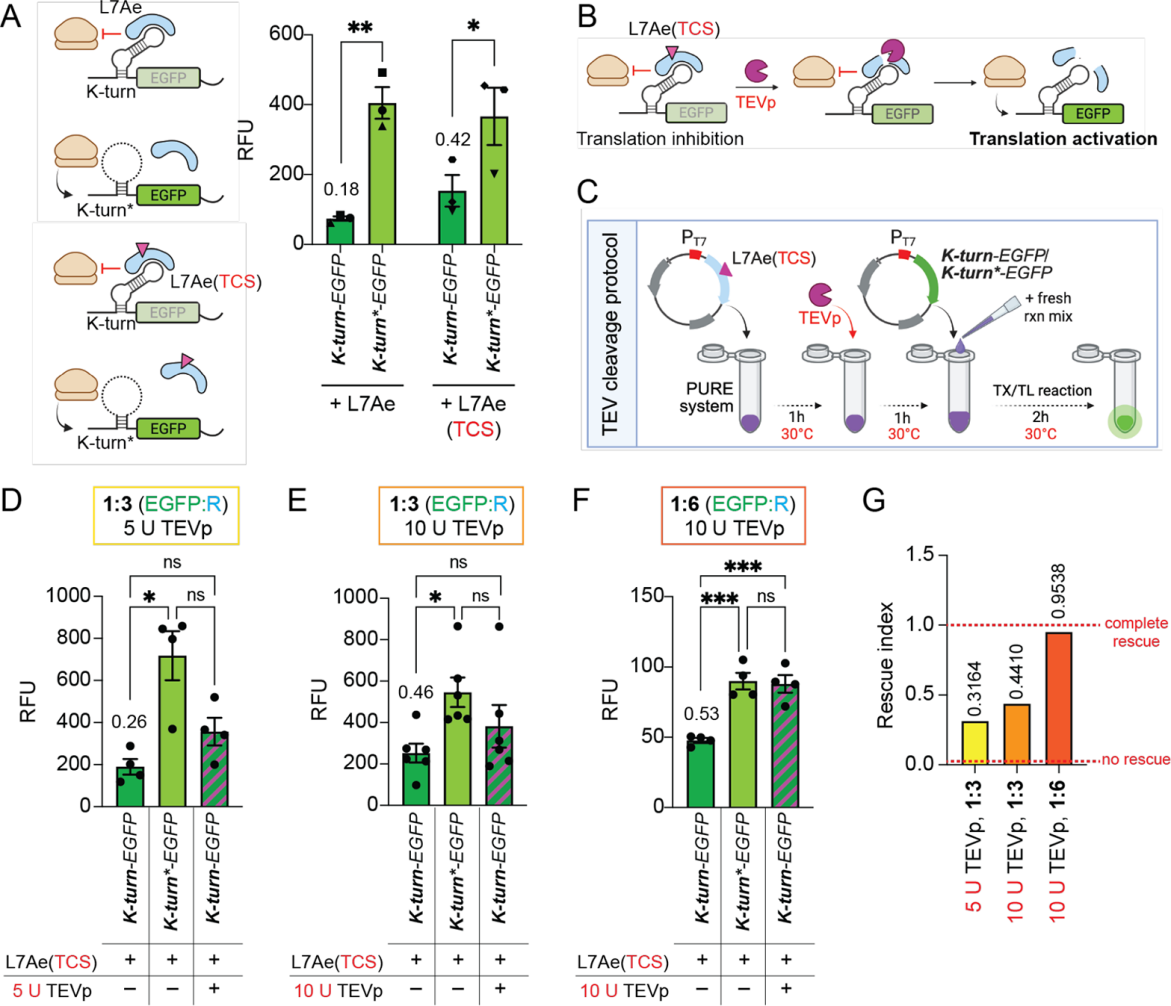
Modulation of *K-turn-EGFP* translation by TEVp-mediated proteolytic inactivation of L7Ae­(TCS) repressor. (A) Schematic of reporter-repressor pairs tested for repression efficiency in PURE upon introduction of the TEV cleavage sequence (TCS) in L7A CDS. Bar plots show mean (±s.e.m.) EGFP expression levels for the indicated constructs. Statistical significance was computed with a one-tailed paired *t* test (*n* = 3). (B) Schematic of TEVp-mediated regulation of L7Ae (TCS) repression activity. *K-turn-EGFP* RNA reporter is translationally repressed by L7Ae (TCS) binding to the translation initiation site. The introduction of TEVp causes the proteolytic cleavage of L7Ae by TCS recognition, releasing *K-turn-EGFP* from the inhibitory state and allowing its translational activation. (C) TEV cleavage protocol in PURE optimized on protocol variant 2. The whole reaction was performed at 30 °C, and recombinant purified TEVp was added in the reaction mix 1 h following L7A/L7Ae (TCS) preincubation. (D–F) L7Ae­(TCS)-mediated repression of wild-type (k-turn) or mutated (k-turn*) EGFP reporters with or without the addition of 5 U (D) or 10 U (E,F) of TEVp. The reporter:repressor (EGFP:R) plasmid molar proportion was 1:3 (D, E) or 1:6 (F). Bar plots show mean (±s.e.m.) EGFP expression levels for the indicated constructs (*n* = 3). Statistical significance was computed with one-way ANOVA followed by Fisher’s LSD test. (G) Rescue index of each condition in D–F. See Materials and Methods for further details. In panels A, D–F, numbers above bars indicate the expression fold change calculated between *K-turn-EGFP* and K-turn*-*EGFP* conditions. **p < 0.01; *p < 0.05; ns = not significant. RNA regulatory elements (k-turn, k-turn*) and different plasmid proportions (1:3, 1:6) are indicated in bold. RFU = relative fluorescent unit.

## Discussion

In this study, we tested the performance of three different posttranscriptional circuits (RISC:*miRNA*, MS2-CNOT7:*ms2L*, and L7Ae:*k-turn*) and evaluated the robustness of their reconstitution in vitro for their prospective application as reliable biosensors in TX-TL cell-free systems.

The RRL was used to test miRNA sensors produced by IVT. With our IVTL RRL-based platform, 2 out of 3 miRNAs expressed in the nM range (the highly expressed miR-451a and the moderately expressed let-7a) were successfully detected with reporters carrying miRNA TS at either 3′ or 5′ UTR, the latter displaying a better correlation with miRNA concentrations measured by qPCR. By eliciting an siRNA-like response, miRNA-guided cleavage of TS placed in the 5′ UTR likely interferes with translation of the target RNA to a greater extent than 3′ UTR cleavage. In fact, translation of capped RNAs with no polyA (as those that result from 3′ UTR cleavage) is highly efficient in RRL, as observed in the context of MS2-CNOT7:*ms2L* experiments and by others.^58,59^ Instead, translation of uncapped RNAs, resulting from 5′ UTR cleavage, is highly inefficient in this lysate,^58^ indicating that miRNA sensors carrying 5′ TS would be better suited for biosensing purposes. However, we observed that these RNA-encoded miRNA sensors display several limitations that must be addressed before their implementation as in vitro biosensors. First, miRNA TS in the 5′ UTR could severely reduce the translational potential of the reporter RNA, due to the possible formation of RNA secondary structures in the proximity of the translation initiation site. This possibility might explain the low luminescence intensities observed in the case of miR-221 5′ TS reporter in all conditions, considering that miR-221 is present in the RRL in the picomolar range as observed by qPCR. Second, miRNA sensors produced by IVT are highly susceptible to RNA degradation due to the unintended introduction of RNases, resulting in false-positive results when their reporter expression is compared to a control RNA. Although extensive RNA degradation could be minimized by operating under RNase-free conditions and by inspecting the prepared RNAs by denaturing gel electrophoresis, even small differences in RNA degradation could impact the final reporter output. To overcome this bias, internal controls such as α-miRNAs are better suited to determine the baseline of each miRNA sensor. However, the functionality of each α-miRNA should be robustly validated prior to its use in IVTL reactions, a procedure that is still suffering its own limitations.^71^ Therefore, proper multilevel control conditions must be carefully designed and introduced in the IVTL miRNA sensing strategy to draw meaningful conclusions on the degree of the specific miRNA-mediated downmodulation effect on the RNA sensor.

The RRL was also used as a eukaryotic cell-free chassis to test the MS2-CNOT7:*ms2L* circuit. In mammalian cells, MS2-CNOT7 and its protease-responsive engineered variants efficiently repress the expression of target ms2L-carrying reporters^31,32^ and display signal rescue using orthogonal proteases.^31^ Although these are promising features for the development of an in vitro biosensor, we found that CNOT7-mediated repression displays major bottlenecks due to counteracting contributions to the modulation of translation of RNAs, hampering its further biosensor development. In addition to its canonical deadenylase activity that exerts RNA degradation through the removal of the polyA,^60^ CNOT7 (also called CAF1) displays an additional repressive activity as reported by in vivo studies that observed the inhibition of polyA-less RNAs.^36^ Although this dual contribution results in a synergistic inhibitory effect on RNA expression in vivo and in cultured cells, we did not observe the same outcome in the RRL, where polyA-less RNAs are translated more efficiently than their polyadenylated counterparts, as also reported by others.^58,59^ Due to this RRL-specific characteristic, our data suggest that the removal of the polyA by CNOT7 causes the target RNA to be translated more efficiently, resulting in an increase of the reporter signal. However, this effect is counteracted by the polyA-independent inhibitory mechanism of CNOT7 that appears to prevail over its translation-boosting effect, as shown by the differences in reporter expression observed when RNAs encoding CNOT7-less constructs are introduced (Figure 2D). Importantly, the same phenomenon of polyA-independent translation was observed also in other lysates (such as HeLa^59^ and yeast^72^) that had been nuclease-treated to degrade endogenous RNA, indicating that the cap-polyA synergy in boosting translation efficiency of transcripts applies mainly when competitor RNAs are present in the lysate.^72^ Nevertheless, HeLa nuclease-treated lysates may be a more adequate chassis than the RRL to develop protease-responsive MS2-CNOT7:*ms2L* cell-free biosensors, provided that the translation efficiency of polyA-less RNAs in these extracts is further validated as being lower than polyadenylated ones.

We also observed how ms2L(−) reporter RNAs, which should not be tethered to the MS2-CNOT7 chimeric protein, are equally affected by translational inhibition than ms2L­(+) constructs, indicating lack of tethering-guided repression of CNOT7 observed in the cellular context,^31^ a prerequisite for developing prospective protease-responsive cell-free biosensors based on the MS2-CNOT7:*ms2L* system. An explanation of this phenomenon might lie in the absence of compartmentalization and molecular crowding in the RRL that is instead characteristic of the cytoplasmic context, where the physiological CCR4-NOT complex or the engineered MS2-CNOT7:*ms2L* system exerts its function in RNA translational control.^73^ In the cell-free context, MS2-CNOT7 proteins tethered to different ms2L-carrying RNAs might be in close vicinity, such that tethering would not be required anymore for localizing CNOT7 activity to its target RNA. However, we could not exclude that this phenomenon could be due to a concentration-dependent, unspecific effect of CNOT7. In fact, titrating the amount of CNOT7 did not lead to conclusive results about this possibility due to the lack of linearity on the CNOT7-mediated inhibition rate, likely depending on the counteracting effects that CNOT7 exerts on RNA translation explained above. All of these observations jeopardize the use of MS2-CNOT7:*ms2L* as a prospective protease-responsive biosensor unless the dual activity of CNOT7 is mechanistically investigated in more detail.

Although the RRL would be the preferred chassis for the reconstitution of mammalian (MS2-CNOT7:*ms2L*) or eukaryotic (RISC:*miRNA*) posttranscriptional regulatory circuits, we have shown that it suffers several limitations mainly due to the difficulty to control interference by endogenous factors and the emergence of potential biases due to differential RNA degradation. L7Ae:*k-turn*, in contrast, could be expressed and tested in eukaryotic (RRL) or prokaryotic (*E. coli* S30) lysates, as well as the prokaryotic-based recombinant PURE system. In fact, the inhibitory effect of L7Ae RBP on its target RNA carrying k-turn secondary structures at its 5′ end appears chassis-agnostic, since it likely depends on steric obstruction of translation initiation.^74^ Compared to other existing L7Ae:*k-turn* circuits reconstituted in a cell-free context,^33,44^ we improve scalability of the system by encoding both components in plasmids, removing the need of providing the repressor as purified protein. With our refined translation protocol, characterization of backbone impact on gene expression, and optimization of repressive condition by varying TEVp and repressor-encoded plasmid amounts, we reconstituted a robust protease-responsive L7Ae:*k-turn* RNP system in PURE, obtaining up to 95% of conditional protease-dependent rescue of L7Ae inhibition. In particular, the robust protease responsiveness of L7Ae:*k-turn* RNP in the PURE system makes it an ideal protein-based biosensing platform for clinically relevant pathogens where a virus-specific protease, rather than its genome, is detected. Protease sensing displays several advantages compared to nucleic acid–based detection methods, such as the possibility to measure active viral replication, better analyte stability, and, most importantly, robustness against viral evolution and emerging variants.^75^ Although qPCR or more recently developed methods that rely on CRISPR-Cas^12,76^ or toehold switches^2,77^ are considered the most sensitive detection technologies, viral protease detection coupled with various signal amplification modules^78–80^ is gaining increasing attention, and offers promising alternatives for the development of viral biosensors that could be implemented in point-of-care or clinical settings.

Previous studies have reconstituted L7Ae-mediated repression on reporter RNAs (previously purified or produced in the same TX-TL reaction) in PURE^33^ and HeLa^44^ cell-free systems. Interestingly, positioning a k-turn corresponding to the wild-type p1boxC/D in the 5′ UTR of the reporter was shown to cause a reduction in the basal expression level in the absence of L7Ae in TX-TL HeLa lysates.^44^ We observed a similar reduction in the reporter expression by inserting the mutated p1boxC/D* sequence immediately downstream of the start codon, which was rescued upon replacing it with the closely related pboxC/D* sequence. Taken together, these data indicate that p1boxC/D k-turn derivatives (either wild-type or mutated) may lead to low levels of reporter expression in cell-free systems, irrespective of whether they are positioned in the 5′ UTR^44^ or in immediately downstream of the ATG (this study). Importantly, this phenomenon appears to be characteristic of TX-TL reactions, since we did not observe such an imbalanced reporter output in a TL-only system. Likely, these discrepancies originate from the different procedures used to produce the RNA and its subsequent purification, which might significantly impact both the folding and the maintenance of secondary structures.^81^ Considering our observations that the plasmid backbone complexity may additionally influence the repression outcome in TX-TL systems such as the *E. coli* S30 lysate and the PURE, a prediction of the activity of the genetic elements encoded in the DNA/RNA constructs and how they may impact gene expression requires further investigation, as well as in-depth characterization of the contribution of UTR/plasmid length and chassis composition.

In conclusion, our results highlight the strengths and limitations of reconstituting RNP complexes in a cell-free context with the aim of evaluating their potential as prospective in vitro biosensors. With this study, we distinguish RNP systems that require further technical (RISC: *miRNA*) or mechanistic (MS2-CNOT7:*ms2L*) characterization and those that show high potential to be further implemented as stimulus-responsive biosensors (L7Ae:*k-turn*). These results provide a step forward toward the development of protease-responsive cell-free biosensors that can be used in translational settings for the rapid, cost-effective, and simple detection of viral pathogens in human samples.

## Materials and Methods

### Design and Generation of Plasmids and PCR Templates

Relevant insert sequences of the plasmids and DNA templates generated in this study are listed in Tables S1 and S2. Unless otherwise noted, In-fusion Cloning (Takara) was used for all cloning procedures, following the manufacturer’s recommendations. All plasmids used were verified by Sanger or whole plasmid sequencing before each application.

DNA fragments used as in vitro transcription templates (see below) were generated by PCR using primers listed in Table S3 annealing to plasmids carrying a T7 promoter (T7p), the gene of interest (GOI), and a short polyA (30A), hereafter called “IVT plasmids.” IVT plasmids (encoding all constructs used for experiments performed in the RRL) were all generated by inserting the GOI into a multiple cloning site (MCS) between the T7p and the polyA (T7-MCS-polyA destination vector). Firefly (FLuc) or NanoLuc (NLuc) luciferase CDS flanked by XbaI+*Sal*I and NheI+XhoI sequences in their 5′ and 3′ UTR, respectively, were inserted as a GOI for the generation of miRNA sensors. 4x miRNA TS were generated by annealing oligos listed in Table S3 and designed such that the sense strand would be complementary to *hsa-miR-451a* (5′-aaaccguuaccauuacugaguu-3′), *hsa-let-7a-5p* (5′-ugagguaguagguuguauaguu-3′), or *hsa-miR-221-3p* (5′-agcuacauugucugcuggguuuc-3′) targeting miR-451a, let-7a, or miR-221, respectively. 300 pmol single-stranded oligos were first 5′ phosphorylated with T4 polynucleotide kinase (PNK) (NEB) following the manufacturer's recommendations in 20 μL reactions. Ten μL aliquots of each PNK reaction containing sense oligos were mixed with the same volume of PNK reactions containing the complementary antisense oligo and annealed by heating the mix to 95 °C followed by slow cooling to RT to create oligo duplexes. 1:150 dilutions of oligo duplexes were ligated into XbaI+*Sal*I- or NheI+XhoI-digested plasmids with T4 ligase (Takara) to generate reporters carrying 5′ UTR TS and 3′ UTR TS, respectively. anti-miRNA sequences (listed in Table S3) were designed to be complementary to the respective target miRNA and purchased as purified synthetic 2′ O-Methyl RNAs from Integrated DNA Technologies (IDT). The MS2-CNOT7 fusion sequence from Cella et al.^31^ was cloned into the T7-MCS-polyA plasmid using In-Fusion cloning (Takara) between the NotI-XbaI restriction sites. The point mutations K58E and K56D in MS2 reported by Peabody^56^ were introduced with the Q5 Site-Directed Mutagenesis kit (NEB) to generate the mutant MS2*-CNOT7 construct. To generate the *FLuc-ms2L* reporter, the FLuc coding sequence was amplified from the T7-FLuc plasmid (Promega) and inserted into the T7-MCS-polyA destination vector. Eight tandem MS2 loop (8xms2L) sequences from Cella et al.^31^ were cloned downstream of FLuc CDS. MS2- and MS2*-only constructs were generated by PCR amplification of the MS2 domain from MS2-CNOT7 and MS2*-CNOT7 plasmids, respectively, followed by cloning into the T7-MCS-polyA destination vector. L7Ae, L7Ae­(TCS), and k-turn* (p1boxC/D*) sequences were obtained from Cella et al.^31^; k-turn (pboxC/D) was obtained from Saito et al.^33^ All sequences were cloned into the destination vector T7-MCS-polyA (to insert the repressor CDS) or T7-NLuc-polyA (to insert the k-turn) to produce the respective IVT plasmids.

For TX-TL expression in *E. coli* S30 lysate and PURE system, L7Ae, L7Ae*, and L7Ae (TCS) were ordered as gene strings and sequences codon optimized for *E. coli* expression. The mutant L7Ae* CDS sequence was obtained from Saito et al.^33^ The destination vector indicated as “BB1” is a standard pET100-DTOPO vector (GeneArt, Thermo), while the “BB2” backbone derives from the removal of the DHFR CDS sequence from PURExpress kit control vector (NEB) (N3273). The full sequences of BB1 and BB2 backbone vectors are listed in File S1. The wild-type pboxC/D and the mutated p1boxC/D* k-turn sequences were fused upstream of the NLuc luciferase CDS in the BB1 vector to produce the *K-turn-NLuc* and K-turn*-NLuc reporters, respectively, used in Figure 3E. The wild-type pboxC/D and the mutated pboxC/D* k-turn sequences were obtained from Saito et al. (2010) and cloned upstream of EGFP CDS in BB1 and BB2 backbones to produce *K-turn-EGFP* and K-turn*-EGFP reporters, respectively, as well as L7Ae, L7Ae*, and L7Ae (TCS) sequences that were amplified by PCR from previously produced plasmids.

### RNA Extraction and Absolute Quantification of miRNAs in RRL by qPCR

Total RNA was isolated from two 70 μL aliquots of RRL (“RRL1” and “RRL2”) with QIAzol (QIAGEN), and the aqueous phase containing RNAs was separated following the addition of chloroform and centrifugation. miRNAs were purified with the RNA Clean and Concentrator-5 kit (Zymo) according to the manufacturer’s instructions for the purification of small (<200 nt) RNAs and eluted in RNase-free H_2_O. RNA was first quantified by Nanodrop, and the actual composition of the “long” and “short” RNA species was evaluated with the Qubit microRNA Assay kit (Invitrogen) and the Qubit RNA IQ Assay kit (Invitrogen) according to the manufacturer’s recommendations. 560 ng of each of 3 different synthetic miRNAs were used as input for standard cDNA production in 3 different reactions with the Mir-X miRNA First Strand Synthesis kit (Takara) in 10 μL volume, and their performance as standards by qPCR was evaluated with 10-fold serial dilutions of the produced cDNA. qPCR was performed with the TB Green qRT-PCR kit (Takara) according to the manufacturer’s instructions using miRNA-specific F primers listed in Table S3 with a BioRad CFX96 Touch Real-Time PCR Detection System. The synthetic miRNA *Nluc2* was chosen as a preferred standard to quantify the absolute concentration of miR-451a, let-7a, and miR-221 in RRL1 and RRL2 by qPCR following cDNA preparation from 300 ng of the respective RNA, with the same kits used for standard cDNA preparation and qPCR. The concentration of each miRNA in the RNA was extrapolated from the standard curve constructed from known *Nluc2* amounts and reported as the concentration of each miRNA in lysates, accounting for starting RRL volume and RNA extraction/elution volumes.

### In Vitro Transcription

DNA templates for IVT were PCR-amplified from IVT plasmids (see above) using primers that annealed to sequences present in all IVT plasmid backbones, namely the T7 promoter sequence (T7p_Fw) and a reverse primer that could include (PolyA+_Rev) or exclude (PolyA-_Rev) the 30A-polyA sequence (Table S3). Sequences of DNA templates used as inputs for IVT are listed in Table S1. Bands of the correct expected size were gel extracted and eluted in RNase-free H_2_O with a Nucleospin Gel and PCR Clean-up kit (Macherey-Nagel) maintained in RNase-free conditions for RNA production applications to avoid RNase contamination. Capped RNAs used as inputs for RRL IVTL reactions were produced from ∼200 ng PCR-amplified templates using the Anti-Reverse Cap Analog (ARCA) (Ambion) with the MEGAscript T7 Transcription kit (Invitrogen) according to the manufacturer’s instructions with some modifications. RNA was prepared by mixing equal amounts of ATP, CTP, UTP, and 4:1 ARCA:GTP mix in 20 μL reactions incubated at 37 °C for 4 h. The reaction was treated with DNase and the RNA was purified with the MEGAclear Transcription Clean-up kit (Invitrogen) according to the manufacturer’s instructions. Eluted RNA was quantified by Nanodrop and its integrity analyzed on Novex TBE-Urea gels (Invitrogen) stained with SYBR Gold before proceeding with IVTL. Purified RNA aliquots were immediately stored at −80 °C.

### miRNA Sensing in RRL and In Vitro Translation

miRNA sensing was performed in RRL, nuclease-treated (Promega) according to the manufacturer's instructions with some modifications adapted from Ricci et al.^52^ to allow endogenous miRNAs to anneal to the respective target sites on the RNA. miRNA-sensing reactions were assembled on ice with 2/3 RRL (v/v), amino acid mixtures, RNasin RNase inhibitor (Promega), and the appropriate anti-miRNA (final 35 nM) or H_2_0 in 30 μL reactions and incubated at 30 °C for 30 min to allow endogenous miRNA to anneal with the cognate anti-miRNA. Tubes were then placed back on ice, and 0.46 fmol of heat-denatured IVT RNA carrying the respective miRNA TS was added to each reaction. The mix was then incubated for 1 h at 10 °C, 2 min at 20 °C, and 2 min at 25 °C to allow endogenous miRNA to anneal with the cognate TS on the reporter RNA in the absence of translation. Translation was activated by increasing the temperature to 30 °C, and luminescence intensity was measured in a luminometer (GloMax Discover, Promega) after 1 h of translation by adding the appropriate luciferase substrate (D-luciferin for FLuc and Endurazine for NLuc) in lysates distributed into 96-well plates.

### MS2-CNOT7:*ms2L* and L7Ae:*k-turn* RNP System Reconstitution by IVTL in RRL

MS2-CNOT7:*ms2L* and L7Ae:*k-turn* RNP system experiments were performed using nuclease-treated RRL (Promega) according to the manufacturer’s instructions with minor modifications to enable luciferase activity detection. For firefly luciferase (FLuc) reporter assays, D-luciferin was added to a final concentration of 50 μM. For NLuc reporter assays, 0.5 μL of 100× Endurazine (Promega) substrate was added per reaction. Reactions were assembled on ice and adjusted to a final volume of 50 μL with RNase-free H_2_O. For MS2-CNOT7:*ms2L* experiments, 280 fmol of RBP (encoding MS2-CNOT7, MS2*-CNOT7, MS2, or MS2*) IVT RNA was pretranslated for 10 min at 30 °C before the addition of 70 fmol of reporter RNA—encoding *FLuc-ms2L* IVT RNA (polyA+ or polyA−) or control *FLuc* RNA (included in the RRL kit)—after which translation proceeded for an additional 60 min prior to luminescence measurement. For the experiment in Figure S2, translation was monitored in real time with luminescence detection every 60 s. MS2-CNOT7 (and the respective MS2*-CNOT7 control) titration was performed by incubating 70 fmol (1:1 LUC:R), 140 fmol (1:2 LUC:R), or 280 fmol (1:4 LUC:R) for 10 min at 30 °C before addition of 70 fmol of reporter RNA encoding polyA + *FLuc-ms2L* RNA. A condition where only the *FLuc-ms2L* reporter was translated (“H_2_O”) was included to have an indication of the “translation capacity” of each lysate aliquot. Luminescence values obtained in each condition (MS2-CNOT7 or MS2*-CNOT7) were normalized with those of the respective H_2_O condition, and the repression fold change was calculated by dividing the normalized values of MS2-CNOT7 by those of the control MS2*-CNOT7. For L7Ae:*k-turn* experiments, 70 fmol of RBP (encoding L7Ae or L7Ae­(TCS)) IVT RNA was preincubated for 10 min at 30 °C prior to the addition of 0.77 fmol of reporter RNA (encoding *K-turn-NLuc* or K-turn*-NLuc) followed by 60 min translation at 30 °C. Luminescence signals were measured using a GloMax Discover luminometer (Promega) following pipetting the IVTL reaction in white 96-well plates.

### Plasmid Preparations for TX-TL in *E. coli* S30 Lysate and PURE

Cloned plasmids were transformed into *E. coli* TOP10 competent cells by heat-shock transformation. Transformed cells were plated on LB agar containing the appropriate antibiotic for selection and incubated overnight. Single colonies were picked the following day and grown in LB broth supplemented with the appropriate antibiotic for 16 h. Plasmids were purified with EZNA Plasmid DNA Mini kit (Omega Biotek) and verified by Sanger sequencing. Sequence-confirmed clones were subsequently grown in larger culture volumes, and plasmids were extracted with QIAGEN Plasmid Midi or Maxi kits to obtain higher-purity DNA and eluted in nuclease-free H_2_O. Purified plasmids were quantified by Qubit dsDNA (BR) Quantification Assay Kit (Invitrogen) following the manufacturer's recommendations and stored at −20 °C.

### L7Ae:*k-turn* RNP System Reconstitution by TX-TL in *E. coli* S30 Lysate

For the experiments in Figure 3E, each reaction includes 5 μL of a complete amino acid mixture, 20 μL of S30 Premix Without Amino Acids, 15 μL of S30 *E. coli* lysate (Promega L1020), and 0.5 μL of Endurazine (Promega), with the final volume adjusted to 50 μL using nuclease-free water. The prepared master mix is aliquoted into 0.2 mL tubes, and 535 fmol of BB1-encoded L7Ae plasmid is added and preincubated at 37 °C for 10 min. Then, 65 fmol of BB1-encoded reporter (*K-turn-NLuc* or K-turn*-NLuc) plasmid was added to the reaction mix and incubated at 37 °C for 1 h. Luminescence was detected following pipetting each reaction in 96-well plates with a luminometer (GloMax Discover, Promega). For the experiments in Figure 3I,J, each reaction is set as described previously, with 535 fmol of repressor-encoded plasmid (L7Ae or L7Ae*, in BB1 or BB2 backbones, as indicated in each graph) added. The mixture was incubated at 37 °C for 10 min, followed by the addition of 65 fmol of reporter-encoded plasmid (*K-turn-EGFP* or K-turn*-EGFP in BB1 or BB2 backbones, as indicated in each graph) to the reaction and incubated at 37 °C for 4 h. Fluorescence was detected following pipetting each reaction in black 96-well plates with a plate reader (GloMax Discover, Promega) detecting EGFP signal (475/500–550 nm ex/em filters).

### L7Ae:*k-turn* RNP System Reconstitution by TX-TL in PURE

Reactions in PURExpress (E6800, NEB) were prepared according to the manufacturer’s recommendations. Briefly, 5 μL of Solution A was mixed with 3.75 μL of Solution B together with 20 U of RNase inhibitor in 0.2 mL nuclease-free PCR tubes. For experiments performed following the protocol designated as “protocol variant 1,” 75 fmol of BB1- or BB2-encoded repressor (L7Ae or L7Ae*) was added to the mix and incubated at 37 °C for 10 min in a thermocycler followed by the addition of 25 fmol of BB1- or BB2-encoded reporter (*K-turn-EGFP* or K-turn*-EGFP). Reactions (final volume 14.5 μL) were incubated at 37 °C for 120 min in a thermocycler. For experiments performed following the protocol designated as “protocol variant 2”, 75 fmol of BB1- or BB2-encoded repressor (L7Ae or L7Ae*) were added to the mix, volumes adjusted to 12.5 μL with nuclease-free H_2_O and mixed gently. After adjustment, tubes were briefly centrifugated and incubated at 37 °C for 120 min in the thermocycler, followed by the addition of 12.5 μL of fresh reaction mix containing 25 fmol of BB1- or BB2-encoded reporter (*K-turn-EGFP* or K-turn*-EGFP). The mix (final volume 25 μL) was then incubated at 37 °C for 120 min in a thermocycler. The volume of all reactions was adjusted to 30 μL with nuclease-free water before pipetting each sample into a 384-well plate. Fluorescence was detected with a plate reader (GloMax Discover, Promega), detecting the EGFP signal (475/500–550 nm ex/em filters).

The L7Ae/L7Ae* plasmid titration experiments in Figure 4E were performed as described above according to protocol variant 1, with the addition of different amounts (25 fmol, 50 fmol, 100 fmol, or 150 fmol) of BB1- or BB2-encoded repressor (L7Ae or L7Ae*), keeping BB1- or BB2-encoded *K-turn-EGFP* reporter plasmid to a constant amount (25 fmol).

### L7Ae­(TCS):*k-turn* RNP System Reconstitution and TEVp-Mediated Rescue by TX-TL in PURE

The experiments involving L7Ae (TCS) and TEVp addition in Figure 5 were performed according to protocol variant 2 with some modifications. Five μL of Solution A was mixed with 3.75 μL of Solution B together with 20 U of RNase inhibitor in 0.2 mL of nuclease-free PCR tubes. 75 fmol (1:3 EGFP:R condition) or 300 fmol (1:6 EGFP:R condition) of BB2-encoded L7Ae­(TCS) was added to the mix and incubated at 30 °C for 1 h. TEV reaction buffer (1.45 μL) together with 5 or 10 U TEVp (NEB, P8112S) was added to the mix and further incubated for 1 h at 30 °C (final volume 14.5 μL). Then, 25 fmol (1:3 EGFP:R condition) or 50 fmol (1:6 EGFP:R condition) of the BB2-encoded reporter (*K-turn-EGFP* or K-turn*-EGFP) plasmid was mixed with fresh Solution A (5 μL) + Solution B (3.75 μL), and the volume was adjusted to 12.5 μL. The reporter-containing fresh reaction was added to the L7Ae­(TCS)- and TEVp-containing mix (final volume 27 μL) and incubated at 30 °C for 2 h before proceeding with fluorescence detection. Fold changes in Figure 5A and D–F were calculated by dividing the average fluorescence values obtained under the *K-turn-EGFP* condition by those of the control K-turn*-EGFP condition, in the absence of TEV protease.

The LOD for the (1:6 EGFP:R condition) was calculated by building a calibration curve with increasing units (U) of TEVp. The LOD was calculated according to the standard deviation of blank method from ICH Q2­(R2) guidelines^82^:

$$\mathrm{LOD}=\frac{3.3\times \sigma }{S}$$

where σ indicates the standard deviation of blank measurements, and *S* indicates the slope of the calibration curve.

The rescue index (RI) in Figure 5G was calculated as the luminescence resulting from the formula:

$$\mathrm{RI}=\frac{(T+\mathrm{TEV}p)-T}{C-T}$$

where T + TEVp = *K-turn-EGFP*+L7Ae­(TCS)+TEVp condition (5 or 10 U), T = *K-turn-EGFP* + L7Ae­(TCS) condition, C = K-turn*-EGFP+L7Ae­(TCS) condition.

### Software

Schematics were created with BioRender, and figures were assembled with Adobe Illustrator. *In silico* cloning, plasmid analysis, and sequencing alignments were done with SnapGene. qPCR data were generated and analyzed with BioRad CFX Maestro software. *In silico* RNA secondary structure prediction was done with Forna.^83^ Data visualization and statistical analysis were done with GraphPad Prism.

## Supplementary Material





## Data Availability

Plasmid insert sequences used in this study are listed in Table S1. The sequences of DNA templates used to produce IVT RNA are listed in Table S2. The sequences of primers and DNA/RNA oligos used in IVT template generation, miRNA TS, anti-miRNA, and qPCR are listed in Table S3. The full sequences of BB1 and BB2 plasmid backbones are available in File S1. Plasmids generated in this study are available upon request.

## Notes

List of BB1 and BB2 sequences (File S1) (PDF)
Endogenous miRNA sensing in the RRL (Figure S1); time-course translation experiment of 70 fmol FLuc-*ms2L* reporter RNAs with or without the polyA in the RRL (Figure S2); titration of repressor-encoding RNAs in the RRL (Figure S3); *k-turn-NLuc* reporter expression in RRL and *E. coli* S30 lysate (Figure S4); in silico folding prediction of wild-type and mutated p1boxC/D and pboxC/D k-turn RNA secondary structures (Figure S5); expression levels of EGFP reporters carrying wild-type or mutated k-turn^pboxC/D^ cloned in BB1 and BB2 plasmid backbones measured in *E. coli* S30 lysate and PURE (Figure S6); BB1 and BB2 plasmid backbone architectures (Figure S7); L7Ae/L7Ae* plasmid titration in the repression of *K-turn-EGFP* reporter encoded in different backbones (Figure S8); overview of protocol optimization outcome for BB1- and BB2-encoded L7A:*k-turn* RNP system in PURE (Figure S9); testing the effect of TEVp buffer, reaction temperature, and reporter:repressor molar ratio on L7Ae­(TCS):*k-turn* system in PURE (Figure S10); sequences of DNA templates used to produce IVT RNA for RRL IVTL (Table S1); DNA Insert sequences in plasmids used for TX-TL reactions (Table S2); oligos and primers used in this study (Table S3) (PDF)

## †

Department of Chemical Sciences and Technologies, University of Rome, Tor Vergata, Via della Ricerca Scientifica 1, 00133 Rome, Italy
