# Supplementary material for: Systematic Evaluation of Different Ribonucleoprotein Complexes as Posttranscriptional Biosensors in Cell-Free TX-TL Systems
Source: ACS Synth Biol. 2026 May 5;15(5):2134–49. doi: 10.1021/acssynbio.6c00170 (PMC13185155; doi:10.1021/acssynbio.6c00170)
Supplement: Supplementary file 2 [file sb6c00170_si_002.pdf]

Supporting Information

Systematic evaluation of different ribonucleoprotein complexes as post-transcriptional biosensors in cell-free TX-TL systems

Dudu Boyvat[1], Francesco Grassi[1#], Lucia Cassella[1†]* and Velia Siciliano[1†]*

[1] Istituto Italiano di Tecnologia-IIT, Synthetic and Systems Biology lab for Biomedicine, Largo Barsanti e Matteucci 53, 80125 Naples, Italy
[#] Present address: Department of Chemical Sciences and Technologies, University of Rome, Tor Vergata, Via della Ricerca Scientifica 1, 00133 Rome, Italy

[†] Equal contribution
* Corresponding authors: Velia Siciliano, velia.siciliano@iit.it; Lucia Cassella, lucia.cassella@iit.it.

Figures S1-S10

Tables S1-S3

Table of content

Figure S1. Endogenous miRNA sensing in the RRL.

Figure S2. Time-course translation experiment of 70 fmol FLuc-*ms2L* reporter RNAs with or without the polyA in the RRL.

Figure S3. Titration of repressor-encoding RNAs in the RRL.

Figure S4. *K-turn-NLuc* reporter expression in RRL and *E. coli* S30 lysate.

Figure S5. *In silico* folding prediction of wild-type and mutated p1boxC/D and pboxC/D k-turn RNA secondary structures.

Figure S6. Expression levels of EGFP reporters carrying wild-type or mutated k-turn$^{pboxC/D}$ cloned in BB1 and BB2 plasmid backbones measured in *E. coli* S30 lysate and PURE.

Figure S7. BB1 and BB2 plasmid backbone architectures.

Figure S8. L7Ae/L7Ae* plasmid titration in the repression of *K-turn-EGFP* reporter encoded in different backbones.

Figure S9. Overview of protocol optimization outcome for BB1- and BB2-encoded L7A:*k-turn* RNP system in PURE.

Figure S10. Testing the effect of TEVp buffer, reaction temperature, and reporter:repressor molar ratio on L7Ae(TCS):*k-turn* system in PURE.

Table S1. Sequences of DNA templates used to produce IVT RNA for RRL IVTL.

Table S2. DNA Insert sequences in plasmids used for TX-TL reactions.

Table S3. Oligos and primers used in this study.

Figure S1

A

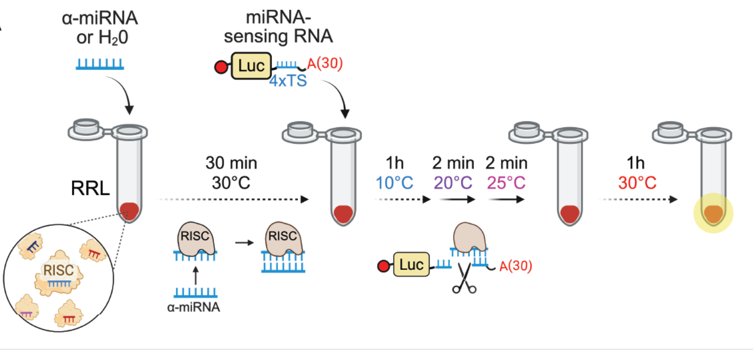

B

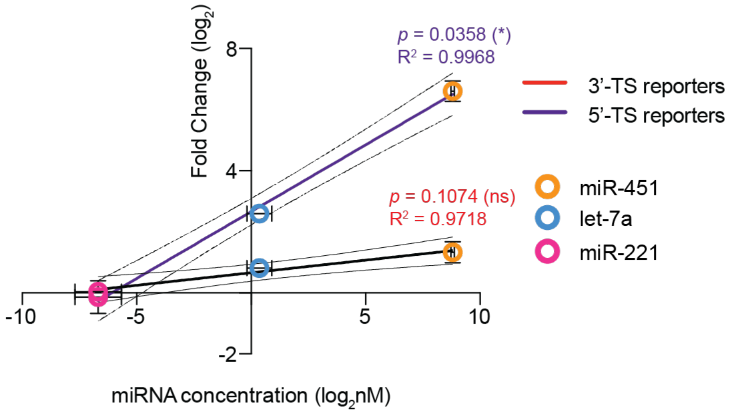

Figure S1. Endogenous miRNA sensing in the RRL. A) Protocol of miRNA sensing in the RRL. An α-miRNA fully complementary to the target miRNA (or H_2O) is incubated in the RRL lysate to allow its annealing with the RISC:*miRNA* complex. The IVT RNA, encoding a luciferase reporter (FLuc or NLuc) and four repetitions of the miRNA TS (displaying the same sequence of the α-miRNA) is then added to the lysate and incubated at low temperature (10 °C, 1h) to allow RISC:*miRNA* annealing to the TS in the absence of translation. Protein synthesis is then activated by a gradual increase of temperature and translation is performed for 1h (30 °C) before luminescence detection. See Materials and Methods for further details. **B)** Correlation analysis of fold change (log_2) measured with 3'-TS or 5'-TS reporters *vs* the concentration of 3 different RRL miRNAs. The nM concentration (log_2-transformed, ± SD, *x* axis) of each indicated miRNA measured by absolute qPCR was plotted against their respective fold changes (log_2-transformed, ± SD, *y* axis) calculated with luciferase-based reporters carrying miRNA TS in their 3'-UTR (red line) or 5'-UTR (violet line). The 95% confidence interval for each regression line is indicated with dashed lines. The coefficient of determination (R^2) and correlation p value (*p*) of each curve is indicated in the respective color above each line.

Figure S2

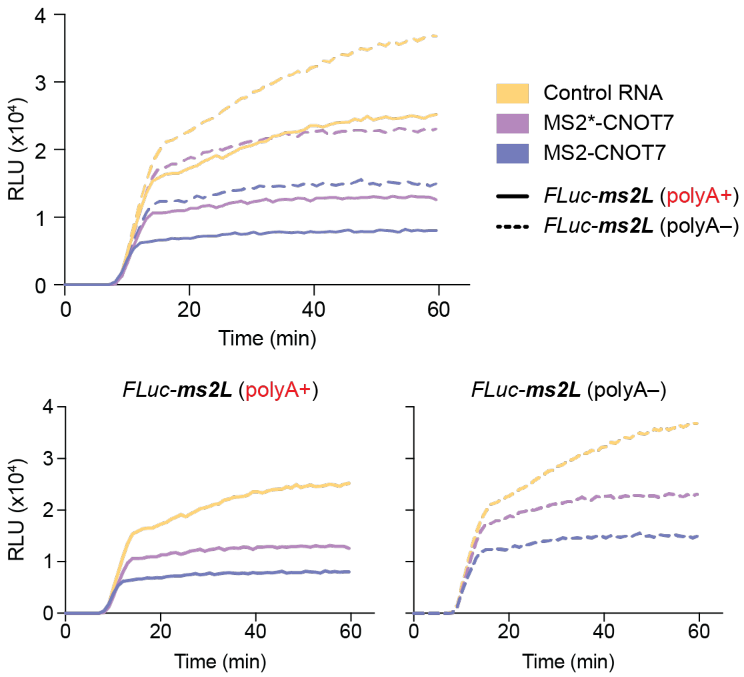

Figure S2. Time-course translation experiment of 70 fmol *FLuc-ms2L* reporter RNAs with (polyA+, solid lines) or without (polyA-, dashed lines) the polyA in the RRL. Each reporter was incubated with RNAs encoding either an “inert“ RNA (Control RNA, yellow) to account for resource consumption, MS2*-CNOT7 (purple), or MS2-CNOT7 (blue). 280 fmol of repressor RNAs were added for each condition. 280 fmol of repressor were converted in the respective ng, and this amount of Control RNA was added to normalize resource consumption across translation reactions containing RNAs of different sizes. RLU=Relative Luminescence Unit.

Figure S3

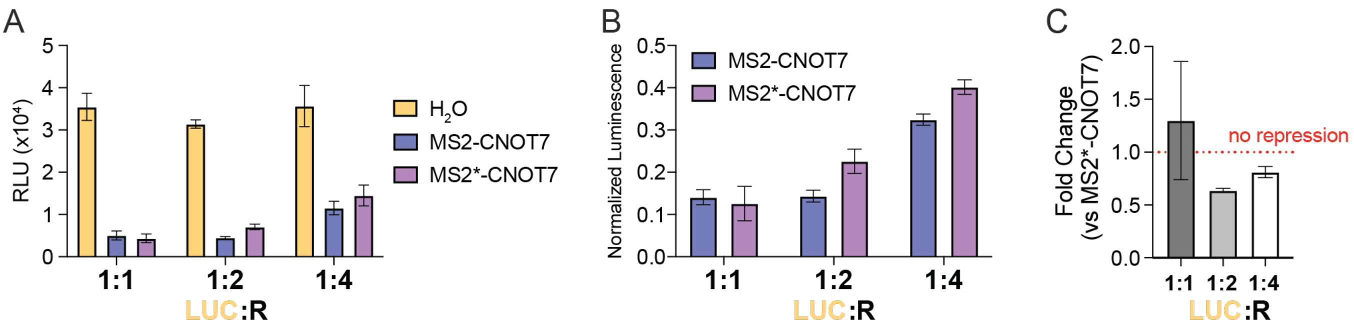

Figure S3. Titration of repressor-encoding RNAs in the RRL. A) Raw luminescence of the indicated samples where the repressor RNA was added to a *FLuc-ms2L*:repressor (LUC:R) ratio of 1:1, 1:2, or 1:4, maintaining the amount of *FLuc-ms2L* RNA constant (70 fmol). Control reactions were set in the absence of the repressor RNA (H_2O, yellow) to control for general lysate translation efficiency across biological replicates. **B)** Luminescence values of the conditions in A normalized to the respective H_2O samples. **C)** Repression fold changes calculated as the ratio between MS-CNOT7 vs. control MS2*-CNOT7 normalized luminescence for the indicated LUC:R conditions. Values below 1 (dashed red line) indicate MS2-CNOT7 mediated repression. Bars show average values ± s.e.m.

Figure S4

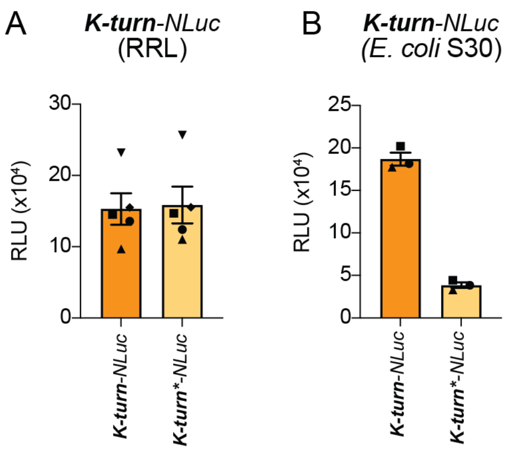

Figure S4. *K-turn-NLuc* reporter expression in RRL and *E. coli* S30 lysate. A) Mean luminescence (± s.e.m) values of NLuc reporters carrying wild-type (k-turn, pboxC/D) and mutated (k-turn*, p1boxC/D*) k-turn secondary structures translated in the RRL (n=5). **B**) Mean luminescence (± s.e.m) values of plasmids encoding the same NLuc reporters as in (A) expressed in the *E. coli* S30 lysate (n=3). RLU=Relative Luminescence Unit.

Figure S5

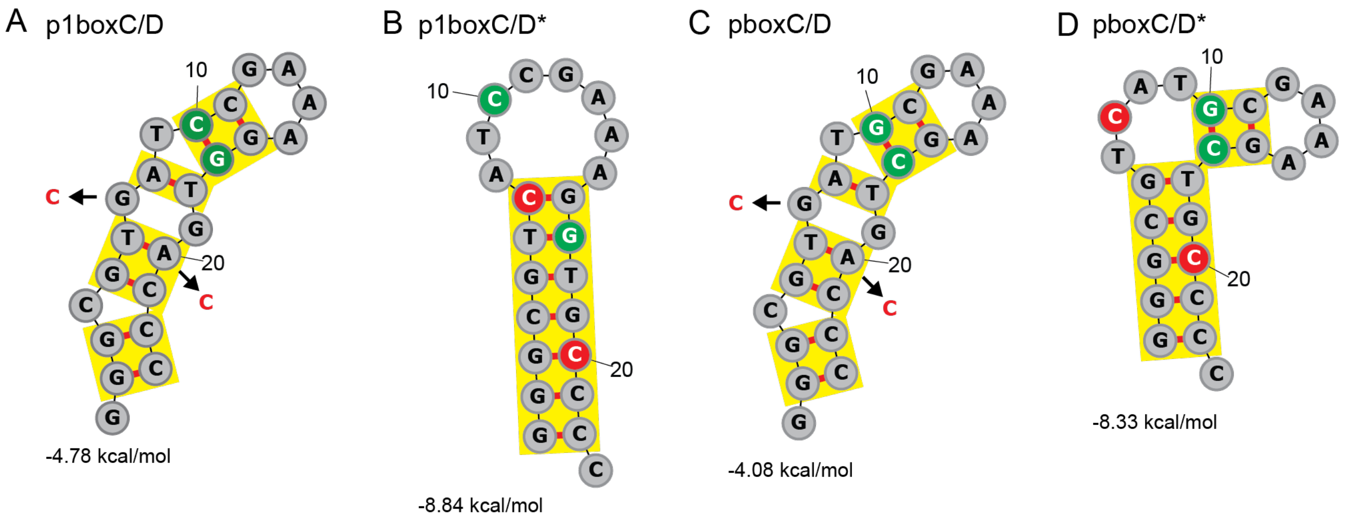

Figure S5. ***In silico* folding prediction of wild-type and mutated p1boxC/D and pboxC/D k-turn RNA secondary structures.** Numbers indicate nucleotide position (5'-3'). Predicted double-stranded regions are highlighted in yellow. Single-nucleotide substitutions generating the respective mutant (k-turn*) are highlighted in red. Nucleotides in green indicate the single base pair difference between p1boxC/D (5'C…G3') and pboxC/D (5'G…C3') and the respective mutant forms. The predicted total free energy is indicated for each secondary structure. **A)** Wild-type (k-turn) p1boxC/D. **B)** Mutated (k-turn*) p1boxC/D. **C)** Wild-type (k-turn) pboxC/D. **D)** Mutated (k-turn*) pboxC/D. Only the k-turn structure indicated in C (pboxC/D) was used as wild-type variant, while the mutant k-turn* structure form was changed from the p1boxC/D* (B) to the pboxC/D* (D) to reduce possible stem-related negative regulation on translation.

Figure S6

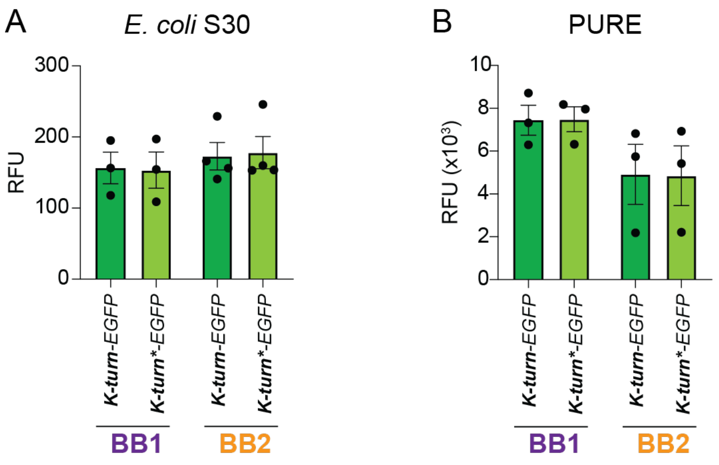

Figure S6. Expression levels of EGFP reporters carrying wild-type or mutated k-turn$^{pboxC/D}$ cloned in BB1 and BB2 plasmid backbones measured in *E. coli* S30 lysate and PURE. Mean fluorescence (± s.e.m) values of EGFP reporters carrying wild-type (k-turn) and mutated (k-turn*) secondary structures (pboxC/D type) encoded in two different backbones (BB1 or BB2) in *E. coli* S30 lysate (A) and in PURE (B). RFU=Relative Fluorescence Unit.

Figure S7

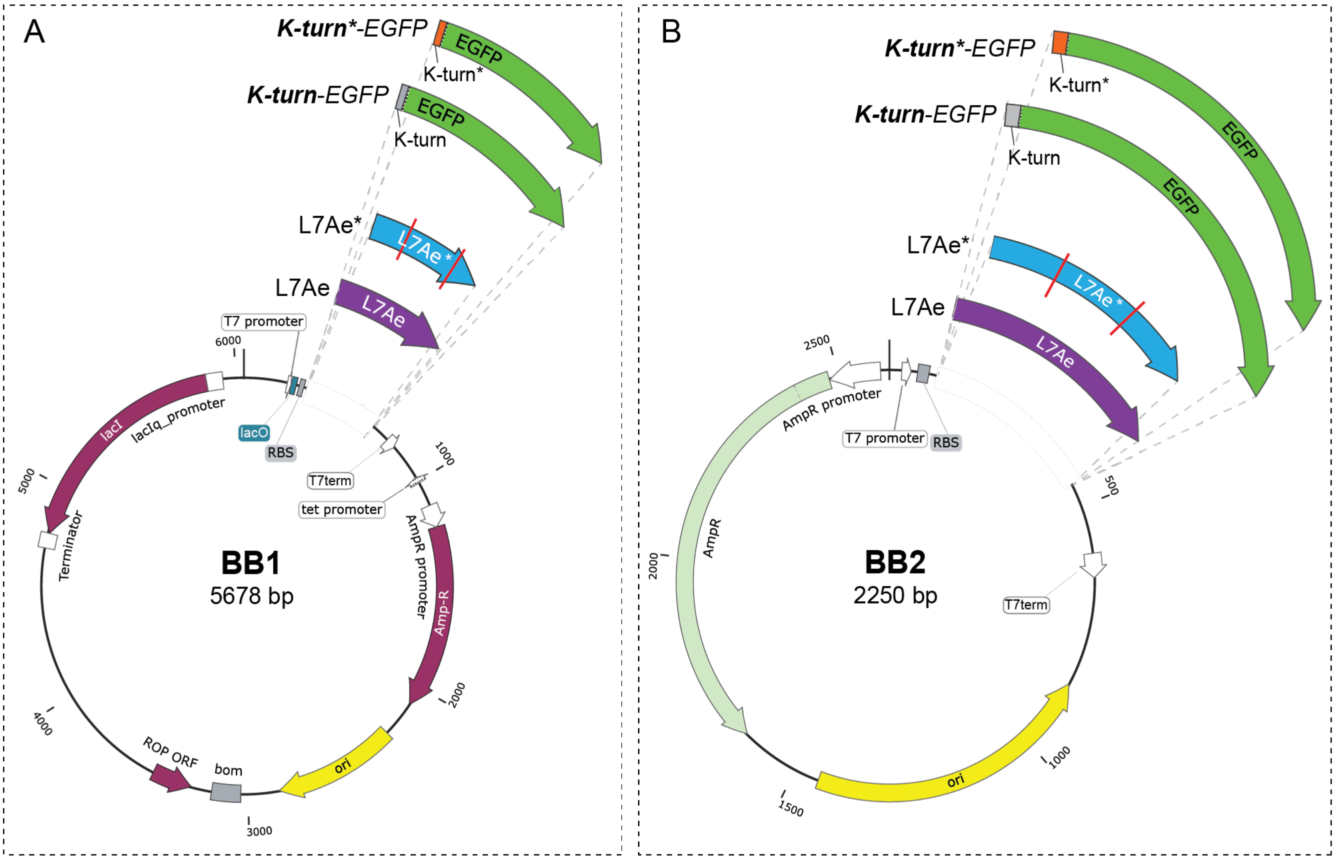

Figure S7. BB1 (A) and BB2 (B) plasmid backbone architectures. Red lines indicate the approximate location of nucleotide sequence variations in L7Ae* CDS with respect to wild-type L7Ae. lacO=lac operator; RBS=Ribosome Binding Site; T7term=T7 terminator; tet=tetracycline; AmpR=ampicillin resistance; ori=origin of replication; bom= basis of mobility; ROP=Rop protein; lacI=Lac Repressor.

Figure S8

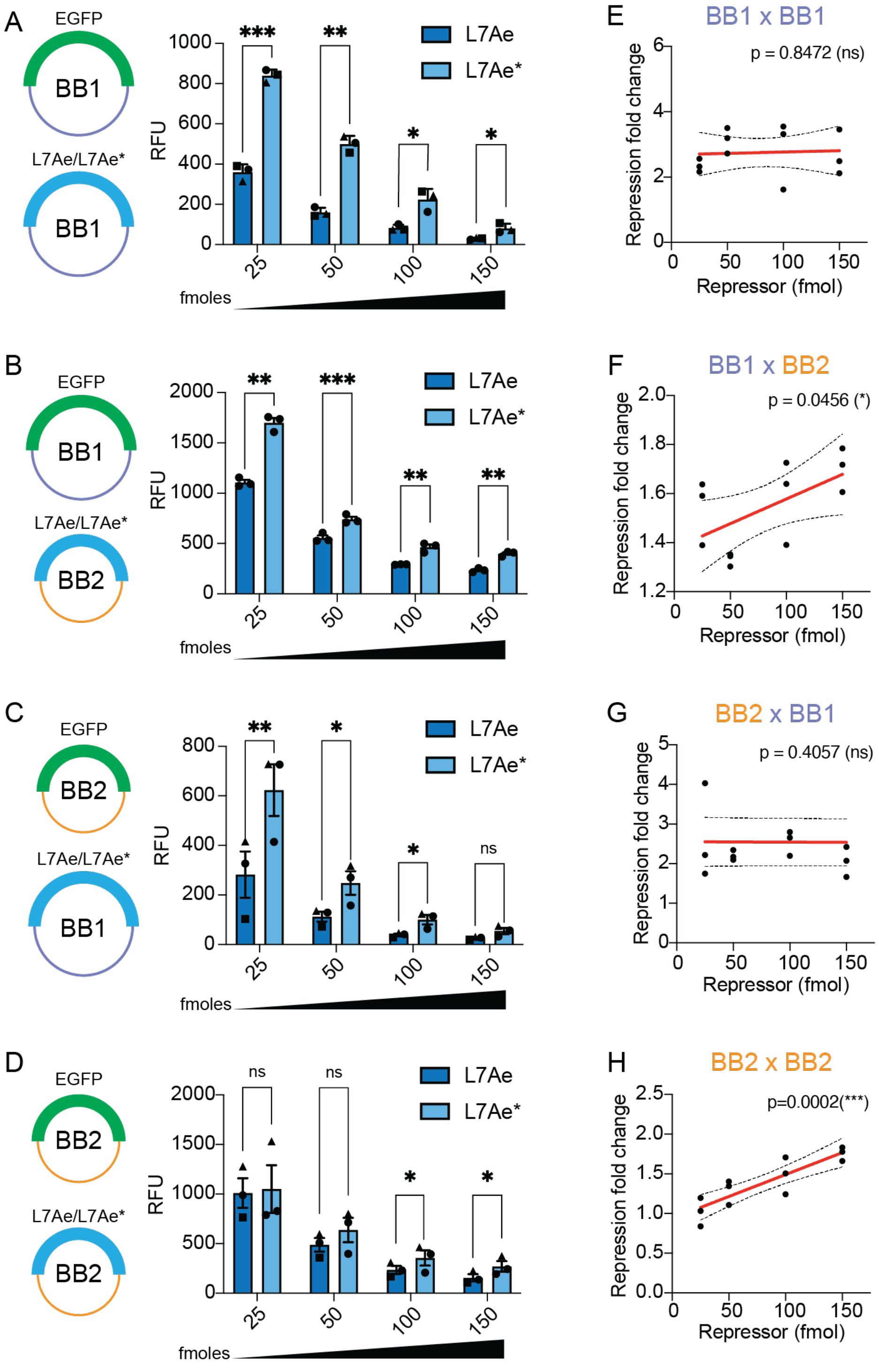

Figure S8. L7Ae/L7Ae* plasmid titration in the repression of *K-turn-EGFP* reporter encoded in different backbones. A-D) Mean (± s.e.m) *K-turn-EGFP* fluorescence with increasing amounts (fmol) of L7Ae or L7Ae*. The plasmid encoding the *K-turn-EGFP* reporter was kept at a constant amount of 25 fmol, while increasing fmol (25, 50, 100, 150) of L7Ae or L7Ae* were added in the pre-incubation step**.** The plasmid backbone encoding the reporter (*K-turn-EGFP*, green) and the repressor (L7Ae/L7Ae*, blue) is indicated on the left. Statistical significance was computed with one-tailed paired t-test (n=3). **E-H**) Simple linear regression of L7Ae repressor fmol (*x* axis) *vs* repression fold change (*y* axis) of L7Ae:*k-turn* RNP circuits encoded in different backbones. In the "BBxBB" abbreviation the first "BB" (BB1 in E-F and BB2 in G-H) is the backbone that carries the reporter; the second "BB" (BB1 in E and G; BB2 in F and H) is the backbone that carries the repressor. The 95% confidence interval for each regression line is indicated with dashed lines. The p value (*p*) is indicated for each regression line (H_0: slope = 0). ***=p<0.001; **= p < 0.01; * = p < 0.05; ns = not significant. RFU= Relative Fluorescence Unit.

Figure S9

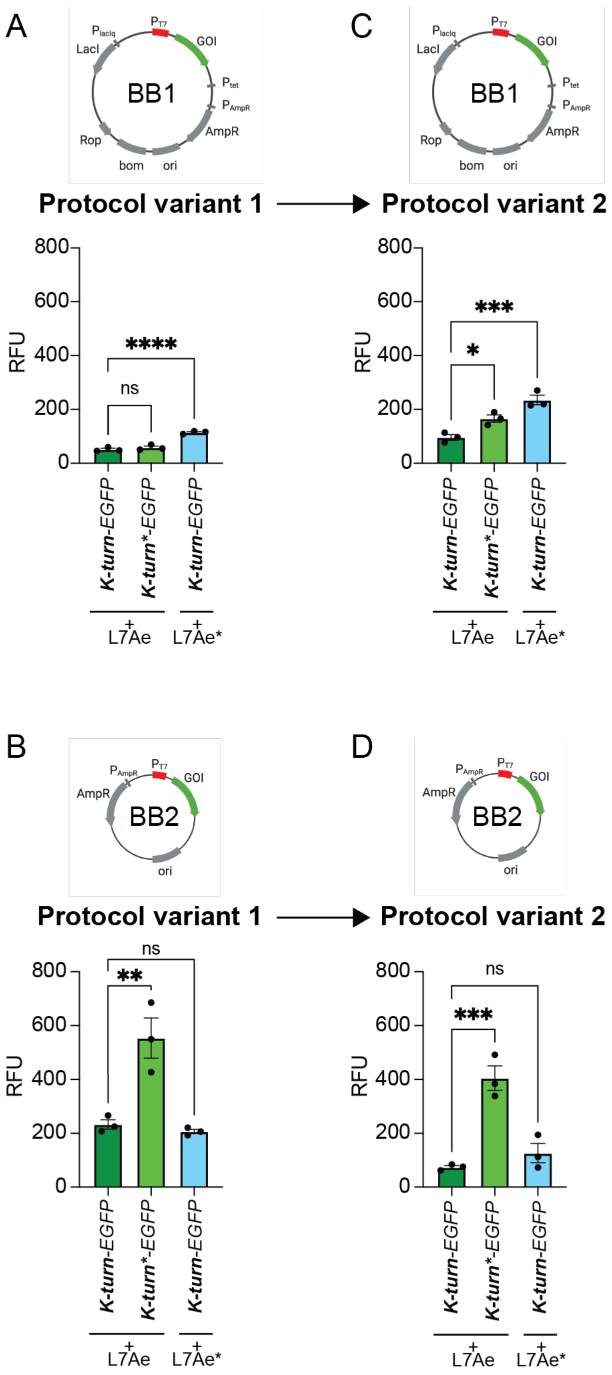

Figure S9. Overview of protocol optimization outcome for BB1- and BB2-encoded L7A:*k-turn* RNP system in PURE. A-B) Mean (± s.e.m) fluorescence of EGFP reporters upon L7Ae repression encoded in BB1 (A) or BB2 (B) backbones with unoptimized translation protocol (protocol variant 1). **C-D)** Mean (± s.e.m) fluorescence of EGFP reporters upon L7Ae repression encoded in BB1 (C) or BB2 (D) backbones following protocol optimization (protocol variant 2). *K-turn-EGFP* or control *K-turn*-EGFP* reporters were incubated with wild-type L7Ae or mutated L7Ae (L7Ae*). Statistical significance was computed with one-way ANOVA followed by Fisher's LSD test (n=3). ***=p<0.001; **= p < 0.01; * = p < 0.05; ns = not significant. RNA regulatory elements (k-turn, k-turn*) are indicated in bold. RFU = Relative Fluorescent Unit.

Figure S10

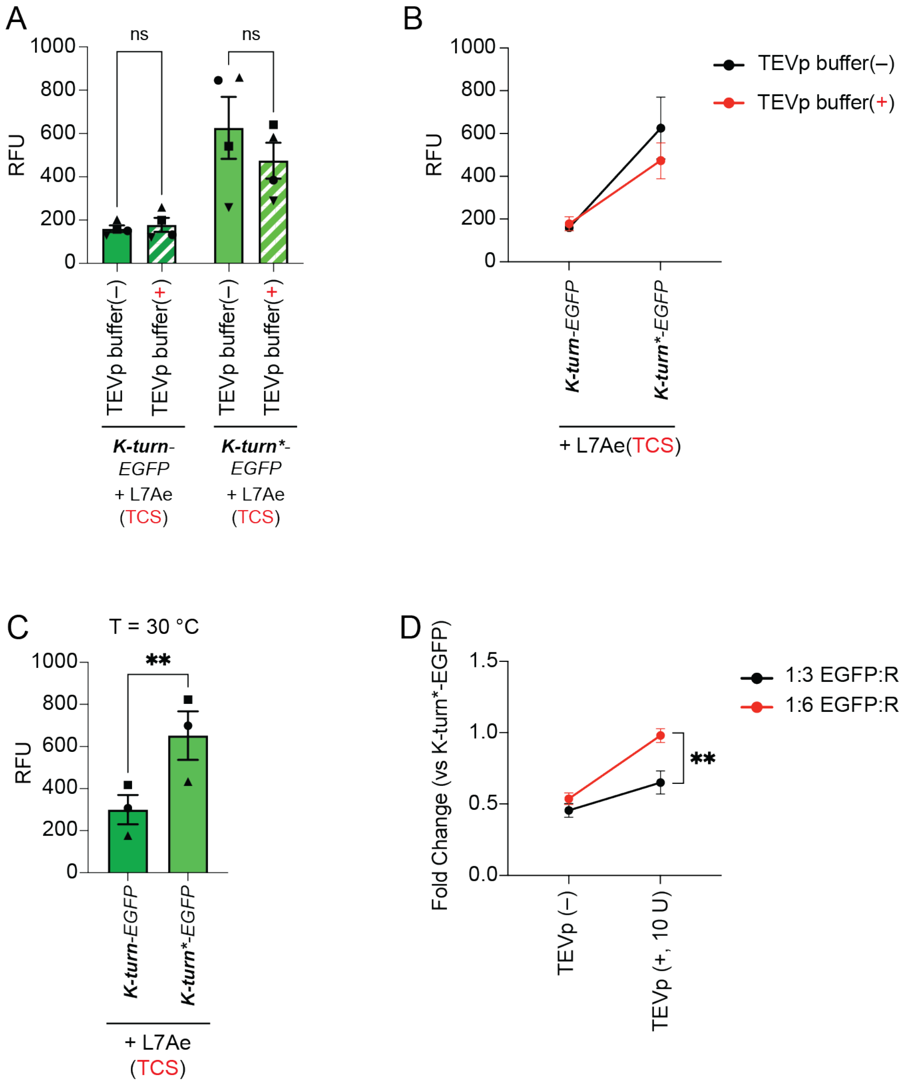

Figure S10. Testing the effect of TEVp buffer, reaction temperature, and reporter:repressor molar ratio on L7Ae(TCS):*k-turn* system in PURE. A-B) Mean fluorescence (± s.e.m) of *K-turn-EGFP* or control *K-turn*-EGFP* reporter upon L7Ae(TCS) repression with or without TEVp buffer. Statistical significance was computed with one-way ANOVA followed by Fisher's LSD test (n=4). **C)** Mean fluorescence (± s.e.m) of *K-turn-EGFP* or control *K-turn*-EGFP* reporter upon L7Ae(TCS) repression of translation reactions carried at 30 °C. Statistical significance was computed with one-tailed paired t test (n=3). **D)** Mean fold change (± s.e.m) of the *K-turn-EGFP* reporter fluorescence upon L7Ae(TCS) repression with or without 10 U of TEVp (summarizing the results reported in Figure 5E-F) relative to the respective *K-turn*-EGFP* negative control. Statistical significance was computed with one-way ANOVA followed by Fisher's LSD test. **= $p < 0.01$; * = $p < 0.05$; ns = not significant. RFU= Relative Fluorescence Unit.

Table S1. Sequences of DNA templates used to produce IVT RNA for RRL IVTL.

<table>
<tr><th>Construct name</th><th>Elements</th><th>Used in Fig</th><th>DNA template sequence (nt)</th></tr>
<tr><td>FLuc-noTS</td><td>T7 promoter
FLuc CDS
3’ polyA (30A)</td><td>1B
1C</td><td>TAATACGACTCACTATAGGGAGACCCAAGCTTATGCATGCGGCCGCATCTAGAGGGCCCGTCGACG
CCACCATGGAAGACGCCAAAAACATAAAGAAAGGCCCGGCGCCATTCTATCCTCTAGAGGATGGAA
CCGCTGGAGAGCAACTGCATAAGGCTATGAAGAGATACGCCCTGGTTCCTGGAACAATTGCTTTTA
CAGATGCACATATCGAGGTGAACATCACGTACGCGGAATACTTCGAAATGTCCGTTCGGTTGGCAG
AAGCTATGAAACGATATGGGCTGAATACAAATCACAGAATCGTCGTATGCAGTGAAAACTCTCTTC
AATTCTTTATGCCGGTGTTGGGCGCGTTATTTATCGGAGTTGCAGTTGCGCCCGCGAACGACATTT
ATAATGAACGTGAATTGCTCAACAGTATGAACATTTCGCAGCCTACCGTAGTGTTTGTTTCCAAAA
AGGGGTTGCAAAAAATTTTGAACGTGCAAAAAAAATTACCAATAATCCAGAAAATTATTATCATGG
ATTCTAAAACGGATTACCAGGGATTTCAGTCGATGTACACGTTCGTCACATCTCATCTACCTCCCG
GTTTTAATGAATACGATTTTGTACCAGAGTCCTTTGATCGTGACAAAACAATTGCACTGATAATGA
ATTCCTCTGGATCTACTGGGTTACCTAAGGGTGTGGCCCTTCCGCATAGAACTGCCTGCGTCAGAT
TCTCGCATGCCAGAGATCCTATTTTTGGCAATCAAATCATTCCGGATACTGCGATTTTAAGTGTTG
TTCCATTCCATCACGGTTTTGGAATGTTTACTACACTCGGATATTTGATATGTGGATTTCGAGTCG
TCTTAATGTATAGATTTGAAGAAGAGCTGTTTTTACGATCCCTTCAGGATTACAAAATTCAAAGTG
CGTTGCTAGTACCAACCCTATTTTCATTCTTCGCCAAAAGCACTCTGATTGACAAATACGATTTAT
CTAATTTACACGAAATTGCTTCTGGGGGCGCACCTCTTTCGAAAGAAGTCGGGGAAGCGGTTGCAA
AACGCTTCCATCTTCCAGGGATACGACAAGGATATGGGCTCACTGAGACTACATCAGCTATTCTGA
TTACACCCGAGGGGGATGATAAACCGGGCGCGGTCGGTAAAGTTGTTCCATTTTTTGAAGCGAAGG
TTGTGGATCTGGATACCGGGAAAACGCTGGGCGTTAATCAGAGAGGCGAATTATGTGTCAGAGGAC
CTATGATTATGTCCGGTTATGTAAACAATCCGGAAGCGACCAACGCCTTGATTGACAAGGATGGAT
GGCTACATTCTGGAGACATAGCTTACTGGGACGAAGACGAACACTTCTTCATAGTTGACCGCTTGA
AGTCTTTAATTAAATACAAAGGATATCAGGTGGCCCCCGCTGAATTGGAATCGATATTGTTACAAC
ACCCCAACATCTTCGACGCGGGCGTGGCAGGTCTTCCCGACGATGACGCCGGTGAACTTCCCGCCG
CCGTTGTTGTTTTGGAGCACGGAAAGACGATGACGGAAAAAGAGATCGTGGATTACGTCGCCAGTC
AAGTAACAACCGCGAAAAAGTTGCGCGGAGGAGTTGTGTTTGTGGACGAAGTACCGAAAGGTCTTA
CCGGAAAACTCGACGCAAGAAAAATCAGAGAGATCCTCATAAAGGCCAAGAAGGGCGGAAAGTCCA
AATTGTAAGGGCTAGCGGATCCCTCGAGGCGATGCGAGACCGGTTAGCTATTGTAATCCTCCGAGG
GGGCGAGCTCCCAAAAAAAAAAAAAAAAAAAAAAAAAAAAAA</td></tr>
<tr><td>FLuc-miR-451</td><td>T7 promoter
FLuc CDS
miR-451a TS (4x)
3’ polyA (30A)</td><td>1B
1C</td><td>TAATACGACTCACTATAGGGAGACCCAAGCTTATGCATGCGGCCGCATCTAGAGGGCCCGTCGACG
CCACCATGGAAGACGCCAAAAACATAAAGAAAGGCCCGGCGCCATTCTATCCTCTAGAGGATGGAA
CCGCTGGAGAGCAACTGCATAAGGCTATGAAGAGATACGCCCTGGTTCCTGGAACAATTGCTTTTA
CAGATGCACATATCGAGGTGAACATCACGTACGCGGAATACTTCGAAATGTCCGTTCGGTTGGCAG
AAGCTATGAAACGATATGGGCTGAATACAAATCACAGAATCGTCGTATGCAGTGAAAACTCTCTTC
AATTCTTTATGCCGGTGTTGGGCGCGTTATTTATCGGAGTTGCAGTTGCGCCCGCGAACGACATTT
ATAATGAACGTGAATTGCTCAACAGTATGAACATTTCGCAGCCTACCGTAGTGTTTGTTTCCAAAA
AGGGGTTGCAAAAAATTTTGAACGTGCAAAAAAAATTACCAATAATCCAGAAAATTATTATCATGG
ATTCTAAAACGGATTACCAGGGATTTCAGTCGATGTACACGTTCGTCACATCTCATCTACCTCCCG
GTTTTAATGAATACGATTTTGTACCAGAGTCCTTTGATCGTGACAAAACAATTGCACTGATAATGA
ATTCCTCTGGATCTACTGGGTTACCTAAGGGTGTGGCCCTTCCGCATAGAACTGCCTGCGTCAGAT
TCTCGCATGCCAGAGATCCTATTTTTGGCAATCAAATCATTCCGGATACTGCGATTTTAAGTGTTG
TTCCATTCCATCACGGTTTTGGAATGTTTACTACACTCGGATATTTGATATGTGGATTTCGAGTCG
TCTTAATGTATAGATTTGAAGAAGAGCTGTTTTTACGATCCCTTCAGGATTACAAAATTCAAAGTG
CGTTGCTAGTACCAACCCTATTTTCATTCTTCGCCAAAAGCACTCTGATTGACAAATACGATTTAT
CTAATTTACACGAAATTGCTTCTGGGGGCGCACCTCTTTCGAAAGAAGTCGGGGAAGCGGTTGCAA
AACGCTTCCATCTTCCAGGGATACGACAAGGATATGGGCTCACTGAGACTACATCAGCTATTCTGA
TTACACCCGAGGGGGATGATAAACCGGGCGCGGTCGGTAAAGTTGTTCCATTTTTTGAAGCGAAGG
TTGTGGATCTGGATACCGGGAAAACGCTGGGCGTTAATCAGAGAGGCGAATTATGTGTCAGAGGAC
CTATGATTATGTCCGGTTATGTAAACAATCCGGAAGCGACCAACGCCTTGATTGACAAGGATGGAT
GGCTACATTCTGGAGACATAGCTTACTGGGACGAAGACGAACACTTCTTCATAGTTGACCGCTTGA
AGTCTTTAATTAAATACAAAGGATATCAGGTGGCCCCCGCTGAATTGGAATCGATATTGTTACAAC
ACCCCAACATCTTCGACGCGGGCGTGGCAGGTCTTCCCGACGATGACGCCGGTGAACTTCCCGCCG
CCGTTGTTGTTTTGGAGCACGGAAAGACGATGACGGAAAAAGAGATCGTGGATTACGTCGCCAGTC
AAGTAACAACCGCGAAAAAGTTGCGCGGAGGAGTTGTGTTTGTGGACGAAGTACCGAAAGGTCTTA
CCGGAAAACTCGACGCAAGAAAAATCAGAGAGATCCTCATAAAGGCCAAGAAGGGCGGAAAGTCCA
AATTGTAAGGGCTAGGGTACC|AACTCAGTAATGGTAACGGTTT|AACTCAGTAATGGTAACGGTTT|
AACTCAGTAATGGTAACGGTTT|AACTCAGTAATGGTAACGGTTT|CCCGGGTCGAGGCGATGCGAGA
CCGGTTAGCTATTGTAATCCTCCGAGGGGGCGAGCTCCCAAAAAAAAAAAAAAAAAAAAAAAAAAA
AAA</td></tr>
<tr><td>FLuc-let-7</td><td>T7 promoter
FLuc CDS
let-7a TS (4x)
3’ polyA (30A)</td><td>1B
1C</td><td>TAATACGACTCACTATAGGGAGACCCAAGCTTATGCATGCGGCCGCATCTAGAGGGCCCGTCGACG
CCACCATGGAAGACGCCAAAAACATAAAGAAAGGCCCGGCGCCATTCTATCCTCTAGAGGATGGAA
CCGCTGGAGAGCAACTGCATAAGGCTATGAAGAGATACGCCCTGGTTCCTGGAACAATTGCTTTTA
CAGATGCACATATCGAGGTGAACATCACGTACGCGGAATACTTCGAAATGTCCGTTCGGTTGGCAG
AAGCTATGAAACGATATGGGCTGAATACAAATCACAGAATCGTCGTATGCAGTGAAAACTCTCTTC
AATTCTTTATGCCGGTGTTGGGCGCGTTATTTATCGGAGTTGCAGTTGCGCCCGCGAACGACATTT
ATAATGAACGTGAATTGCTCAACAGTATGAACATTTCGCAGCCTACCGTAGTGTTTGTTTCCAAAA
AGGGGTTGCAAAAAATTTTGAACGTGCAAAAAAAATTACCAATAATCCAGAAAATTATTATCATGG
ATTCTAAAACGGATTACCAGGGATTTCAGTCGATGTACACGTTCGTCACATCTCATCTACCTCCCG
GTTTTAATGAATACGATTTTGTACCAGAGTCCTTTGATCGTGACAAAACAATTGCACTGATAATGA
ATTCCTCTGGATCTACTGGGTTACCTAAGGGTGTGGCCCTTCCGCATAGAACTGCCTGCGTCAGAT
TCTCGCATGCCAGAGATCCTATTTTTGGCAATCAAATCATTCCGGATACTGCGATTTTAAGTGTTG</td></tr>
</table>

			TTCCATTCCATCACGGTTTTGGAATGTTTACTACACTCGGATATTTGATATGTGGATTTCGAGTCG<br>TCTTAATGTATAGATTTGAAGAAGAGCTGTTTTTACGATCCCTTCAGGATTACAAAATTCAAAGTG<br>CGTTGCTAGTACCAACCCTATTTTCATTCTTCGCCAAAAGCACTCTGATTGACAAATACGATTTAT<br>CTAATTTACACGAAATTGCTTCTGGGGGCGCACCTCTTTCGAAAGAAGTCGGGGAAGCGGTTGCAA<br>AACGCTTCCATCTTCCAGGGATACGACAAGGATATGGGCTCACTGAGACTACATCAGCTATTCTGA<br>TTACACCCGAGGGGGATGATAAACCGGGCGCGGTCGGTAAAGTTGTTCCATTTTTTGAAGCGAAGG<br>TTGTGGATCTGGATACCGGGAAAACGCTGGGCGTTAATCAGAGAGGCGAATTATGTGTCAGAGGAC<br>CTATGATTATGTCCGGTTATGTAAACAATCCGGAAGCGACCAACGCCTTGATTGACAAGGATGGAT<br>GGCTACATTCTGGAGACATAGCTTACTGGGACGAAGACGAACACTTCTTCATAGTTGACCGCTTGA<br>AGTCTTTAATTAAATACAAAGGATATCAGGTGGCCCCCGCTGAATTGGAATCGATATTGTTACAAC<br>ACCCCAACATCTTCGACGCGGGCGTGGCAGGTCTTCCCGACGATGACGCCGGTGAACTTCCCGCCG<br>CCGTTGTTGTTTTGGAGCACGGAAAGACGATGACGGAAAAAGAGATCGTGGATTACGTCGCCAGTC<br>AAGTAACAACCGCGAAAAAGTTGCGCGGAGGAGTTGTGTTTGTGGACGAAGTACCGAAAGGTCTTA<br>CCGGAAAACTCGACGCAAGAAAAATCAGAGAGATCCTCATAAAGGCCAAGAAGGGCGGAAAGTCCA<br>AATTGTAAGGGCTAGGGTACC|AACTATACAACCTACTACCTCA|AACTATACAACCTACTACCTCA| AACTATACAACCTACTACCTCA|AACTATACAACCTACTACCTCA|CCCGGGTCGAGGCGATGCGAGA<br>CCGGTTAGCTATTGTAATCCTCCGAGGGGGCGAGCTCCCAAAAAAAAAAAAAAAAAAAAAAAAAAA<br>AAA
FLuc-miR-221	T7 promoter<br>FLuc CDS<br>miR-221 TS (4x)<br>3’ polyA (30A)	1B<br>1C	**TAATACGACTCACTATAGG**GAGACCCAAGCTTATGCATGCGGCCGCATCTAGAGGGCCCGTCGACG<br>CCACCATGGAAGACGCCAAAAACATAAAGAAAGGCCCGGCGCCATTCTATCCTCTAGAGGATGGAA<br>CCGCTGGAGAGCAACTGCATAAGGCTATGAAGAGATACGCCCTGGTTCCTGGAACAATTGCTTTTA<br>CAGATGCACATATCGAGGTGAACATCACGTACGCGGAATACTTCGAAATGTCCGTTCGGTTGGCAG<br>AAGCTATGAAACGATATGGGCTGAATACAAATCACAGAATCGTCGTATGCAGTGAAAACTCTCTTC<br>AATTCTTTATGCCGGTGTTGGGCGCGTTATTTATCGGAGTTGCAGTTGCGCCCGCGAACGACATTT<br>ATAATGAACGTGAATTGCTCAACAGTATGAACATTTCGCAGCCTACCGTAGTGTTTGTTTCCAAAA<br>AGGGGTTGCAAAAAATTTTGAACGTGCAAAAAAAATTACCAATAATCCAGAAAATTATTATCATGG<br>ATTCTAAAACGGATTACCAGGGATTTCAGTCGATGTACACGTTCGTCACATCTCATCTACCTCCCG<br>GTTTTAATGAATACGATTTTGTACCAGAGTCCTTTGATCGTGACAAAACAATTGCACTGATAATGA<br>ATTCCTCTGGATCTACTGGGTTACCTAAGGGTGTGGCCCTTCCGCATAGAACTGCCTGCGTCAGAT<br>TCTCGCATGCCAGAGATCCTATTTTTGGCAATCAAATCATTCCGGATACTGCGATTTTAAGTGTTG<br>TTCCATTCCATCACGGTTTTGGAATGTTTACTACACTCGGATATTTGATATGTGGATTTCGAGTCG<br>TCTTAATGTATAGATTTGAAGAAGAGCTGTTTTTACGATCCCTTCAGGATTACAAAATTCAAAGTG<br>CGTTGCTAGTACCAACCCTATTTTCATTCTTCGCCAAAAGCACTCTGATTGACAAATACGATTTAT<br>CTAATTTACACGAAATTGCTTCTGGGGGCGCACCTCTTTCGAAAGAAGTCGGGGAAGCGGTTGCAA<br>AACGCTTCCATCTTCCAGGGATACGACAAGGATATGGGCTCACTGAGACTACATCAGCTATTCTGA<br>TTACACCCGAGGGGGATGATAAACCGGGCGCGGTCGGTAAAGTTGTTCCATTTTTTGAAGCGAAGG<br>TTGTGGATCTGGATACCGGGAAAACGCTGGGCGTTAATCAGAGAGGCGAATTATGTGTCAGAGGAC<br>CTATGATTATGTCCGGTTATGTAAACAATCCGGAAGCGACCAACGCCTTGATTGACAAGGATGGAT<br>GGCTACATTCTGGAGACATAGCTTACTGGGACGAAGACGAACACTTCTTCATAGTTGACCGCTTGA<br>AGTCTTTAATTAAATACAAAGGATATCAGGTGGCCCCCGCTGAATTGGAATCGATATTGTTACAAC<br>ACCCCAACATCTTCGACGCGGGCGTGGCAGGTCTTCCCGACGATGACGCCGGTGAACTTCCCGCCG<br>CCGTTGTTGTTTTGGAGCACGGAAAGACGATGACGGAAAAAGAGATCGTGGATTACGTCGCCAGTC<br>AAGTAACAACCGCGAAAAAGTTGCGCGGAGGAGTTGTGTTTGTGGACGAAGTACCGAAAGGTCTTA<br>CCGGAAAACTCGACGCAAGAAAAATCAGAGAGATCCTCATAAAGGCCAAGAAGGGCGGAAAGTCCA<br>AATTGTAAGGGCTAGGGTACC|GAAACCCAGCAGACAATGTAGCT|GAAACCCAGCAGACAATGTAGC<br>T|GAAACCCAGCAGACAATGTAGCT|GAAACCCAGCAGACAATGTAGCT|CCCGGGTCGAGGCGATGC<br>GAGACCGGTTAGCTATTGTAATCCTCCGAGGGGGCGAGCTCCCAAAAAAAAAAAAAAAAAAAAAAA<br>AAAAAAA
NLuc-miR-451	T7 promoter<br>NLuc CDS<br>miR-451a TS (4x)<br>3’ polyA (30A)	1E<br>S1	**TAATACGACTCACTATAGG**GAGACCCAAGCTTATGCATGCGGCCGCATCTAGAGGGCCCGTCGACG<br>CCACCATGGTCTTCACACTCGAAGATTTCGTTGGGGACTGGCGACAGACAGCCGGCTACAACCTGG<br>ACCAAGTCCTTGAACAGGGAGGTGTGTCCAGTTTGTTTCAGAATCTCGGGGTGTCCGTAACTCCGA<br>TCCAAAGGATTGTCCTGAGCGGTGAAAATGGGCTGAAGATCGACATCCATGTCATCATCCCGTATG<br>AAGGTCTGAGCGGCGACCAAATGGGCCAGATCGAAAAAATTTTTAAGGTGGTGTACCCTGTGGATG<br>ATCATCACTTTAAGGTGATCCTGCACTATGGCACACTGGTAATCGACGGGGTTACGCCGAACATGA<br>TCGACTATTTCGGACGGCCGTATGAAGGCATCGCCGTGTTCGACGGCAAAAAGATCACTGTAACAG<br>GGACCCTGTGGAACGGCAACAAAATTATCGACGAGCGCCTGATCAACCCCGACGGCTCCCTGCTGT<br>TCCGAGTAACCATCAACGGAGTGACCGGCTGGCGGCTGTGCGAACGCATTCTGGCGTAAGGGCTAG<br>GGTACC|AACTCAGTAATGGTAACGGTTT|AACTCAGTAATGGTAACGGTTT|AACTCAGTAATGGTA<br>ACGGTTT|AACTCAGTAATGGTAACGGTTT|CCCGGGTCGAGTAGCTATTGTAATCCTCCGAGGGGGC<br>GAGCTCCCAAAAAAAAAAAAAAAAAAAAAAAAAAAAAA
NLuc-let-7	T7 promoter<br>NLuc CDS<br>let-7a TS (4x)<br>3’ polyA (30A)	1E<br>S1	**TAATACGACTCACTATAGG**GAGACCCAAGCTTATGCATGCGGCCGCATCTAGAGGGCCCGTCGACG<br>CCACCATGGTCTTCACACTCGAAGATTTCGTTGGGGACTGGCGACAGACAGCCGGCTACAACCTGG<br>ACCAAGTCCTTGAACAGGGAGGTGTGTCCAGTTTGTTTCAGAATCTCGGGGTGTCCGTAACTCCGA<br>TCCAAAGGATTGTCCTGAGCGGTGAAAATGGGCTGAAGATCGACATCCATGTCATCATCCCGTATG<br>AAGGTCTGAGCGGCGACCAAATGGGCCAGATCGAAAAAATTTTTAAGGTGGTGTACCCTGTGGATG<br>ATCATCACTTTAAGGTGATCCTGCACTATGGCACACTGGTAATCGACGGGGTTACGCCGAACATGA<br>TCGACTATTTCGGACGGCCGTATGAAGGCATCGCCGTGTTCGACGGCAAAAAGATCACTGTAACAG<br>GGACCCTGTGGAACGGCAACAAAATTATCGACGAGCGCCTGATCAACCCCGACGGCTCCCTGCTGT<br>TCCGAGTAACCATCAACGGAGTGACCGGCTGGCGGCTGTGCGAACGCATTCTGGCGTAAGGGCTAG<br>GGTACC|AACTATACAACCTACTACCTCA|AACTATACAACCTACTACCTCA|AACTATACAACCTAC

<table>
<tr><td></td><td></td><td></td><td>TACCTCA|AACTATACAACCTACTACCTCA|CCCGGGTCGAGTAGCTATTGTAATCCTCCGAGGGGGC
GAGCTCCCAAAAAAAAAAAAAAAAAAAAAAAAAAAAAA</td></tr>
<tr><td>NLuc-miR-221</td><td>T7 promoter
NLuc CDS
miR-221 TS (4x)
3’ polyA (30A)</td><td>1E
S1</td><td>TAATACGACTCACTATAGGGAGACCCAAGCTTATGCATGCGGCCGCATCTAGAGGGCCCGTCGACG
CCACCATGGTCTTCACACTCGAAGATTTCGTTGGGGACTGGCGACAGACAGCCGGCTACAACCTGG
ACCAAGTCCTTGAACAGGGAGGTGTGTCCAGTTTGTTTCAGAATCTCGGGGTGTCCGTAACTCCGA
TCCAAAGGATTGTCCTGAGCGGTGAAAATGGGCTGAAGATCGACATCCATGTCATCATCCCGTATG
AAGGTCTGAGCGGCGACCAAATGGGCCAGATCGAAAAAATTTTTAAGGTGGTGTACCCTGTGGATG
ATCATCACTTTAAGGTGATCCTGCACTATGGCACACTGGTAATCGACGGGGTTACGCCGAACATGA
TCGACTATTTCGGACGGCCGTATGAAGGCATCGCCGTGTTCGACGGCAAAAAGATCACTGTAACAG
GGACCCTGTGGAACGGCAACAAAATTATCGACGAGCGCCTGATCAACCCCGACGGCTCCCTGCTGT
TCCGAGTAACCATCAACGGAGTGACCGGCTGGCGGCTGTGCGAACGCATTCTGGCGTAAGGGCTAG
GGTACC|GAAACCCAGCAGACAATGTAGCT|GAAACCCAGCAGACAATGTAGCT|GAAACCCAGCAGA
CAATGTAGCT|GAAACCCAGCAGACAATGTAGCT|CCCGGGTCGAGTAGCTATTGTAATCCTCCGAGG
GGGCGAGCTCCCAAAAAAAAAAAAAAAAAAAAAAAAAAAAAA</td></tr>
<tr><td>miR-451-NLuc</td><td>T7 promoter
miR-451a TS (4x)
NLuc CDS
3’ polyA (30A)</td><td>1F
S1</td><td>TAATACGACTCACTATAGGGAGACCCAAGCTTATGCATGCGGCCGCATCTAGGGTACC|AACTCAGT
AATGGTAACGGTTT|AACTCAGTAATGGTAACGGTTT|AACTCAGTAATGGTAACGGTTT|AACTCAG
TAATGGTAACGGTTT|CCCGGGTCGACGCCACCATGGTCTTCACACTCGAAGATTTCGTTGGGGACT
GGCGACAGACAGCCGGCTACAACCTGGACCAAGTCCTTGAACAGGGAGGTGTGTCCAGTTTGTTTC
AGAATCTCGGGGTGTCCGTAACTCCGATCCAAAGGATTGTCCTGAGCGGTGAAAATGGGCTGAAGA
TCGACATCCATGTCATCATCCCGTATGAAGGTCTGAGCGGCGACCAAATGGGCCAGATCGAAAAAA
TTTTTAAGGTGGTGTACCCTGTGGATGATCATCACTTTAAGGTGATCCTGCACTATGGCACACTGG
TAATCGACGGGGTTACGCCGAACATGATCGACTATTTCGGACGGCCGTATGAAGGCATCGCCGTGT
TCGACGGCAAAAAGATCACTGTAACAGGGACCCTGTGGAACGGCAACAAAATTATCGACGAGCGCC
TGATCAACCCCGACGGCTCCCTGCTGTTCCGAGTAACCATCAACGGAGTGACCGGCTGGCGGCTGT
GCGAACGCATTCTGGCGTAAGGGCTAGCGGATCCCTCGAGTAGCTATTGTAATCCTCCGAGGGGGC
GAGCTCCCAAAAAAAAAAAAAAAAAAAAAAAAAAAAAA</td></tr>
<tr><td>let-7-NLuc</td><td>T7 promoter
let-7a TS (4x)
NLuc CDS
3’ polyA (30A)</td><td>1F
S1</td><td>TAATACGACTCACTATAGGGAGACCCAAGCTTATGCATGCGGCCGCATCTAGGGTACC|AACTATAC
AACCTACTACCTCA|AACTATACAACCTACTACCTCA|AACTATACAACCTACTACCTCA|AACTATA
CAACCTACTACCTCA|CCCGGGTCGACGCCACCATGGTCTTCACACTCGAAGATTTCGTTGGGGACT
GGCGACAGACAGCCGGCTACAACCTGGACCAAGTCCTTGAACAGGGAGGTGTGTCCAGTTTGTTTC
AGAATCTCGGGGTGTCCGTAACTCCGATCCAAAGGATTGTCCTGAGCGGTGAAAATGGGCTGAAGA
TCGACATCCATGTCATCATCCCGTATGAAGGTCTGAGCGGCGACCAAATGGGCCAGATCGAAAAAA
TTTTTAAGGTGGTGTACCCTGTGGATGATCATCACTTTAAGGTGATCCTGCACTATGGCACACTGG
TAATCGACGGGGTTACGCCGAACATGATCGACTATTTCGGACGGCCGTATGAAGGCATCGCCGTGT
TCGACGGCAAAAAGATCACTGTAACAGGGACCCTGTGGAACGGCAACAAAATTATCGACGAGCGCC
TGATCAACCCCGACGGCTCCCTGCTGTTCCGAGTAACCATCAACGGAGTGACCGGCTGGCGGCTGT
GCGAACGCATTCTGGCGTAAGGGCTAGCGGATCCCTCGAGTAGCTATTGTAATCCTCCGAGGGGGC
GAGCTCCCAAAAAAAAAAAAAAAAAAAAAAAAAAAAAA</td></tr>
<tr><td>miR-221-NLuc</td><td>T7 promoter
miR-221 TS (4x)
NLuc CDS
3’ polyA (30A)</td><td>1F
S1</td><td>TAATACGACTCACTATAGGGAGACCCAAGCTTATGCATGCGGCCGCATCTAGGGTACC|GAAACCCA
GCAGACAATGTAGCT|GAAACCCAGCAGACAATGTAGCT|GAAACCCAGCAGACAATGTAGCT|GAAA
CCCAGCAGACAATGTAGCT|CCCGGGTCGACGCCACCATGGTCTTCACACTCGAAGATTTCGTTGGG
GACTGGCGACAGACAGCCGGCTACAACCTGGACCAAGTCCTTGAACAGGGAGGTGTGTCCAGTTTG
TTTCAGAATCTCGGGGTGTCCGTAACTCCGATCCAAAGGATTGTCCTGAGCGGTGAAAATGGGCTG
AAGATCGACATCCATGTCATCATCCCGTATGAAGGTCTGAGCGGCGACCAAATGGGCCAGATCGAA
AAAATTTTTAAGGTGGTGTACCCTGTGGATGATCATCACTTTAAGGTGATCCTGCACTATGGCACA
CTGGTAATCGACGGGGTTACGCCGAACATGATCGACTATTTCGGACGGCCGTATGAAGGCATCGCC
GTGTTCGACGGCAAAAAGATCACTGTAACAGGGACCCTGTGGAACGGCAACAAAATTATCGACGAG
CGCCTGATCAACCCCGACGGCTCCCTGCTGTTCCGAGTAACCATCAACGGAGTGACCGGCTGGCGG
CTGTGCGAACGCATTCTGGCGTAAGGGCTAGCGGATCCCTCGAGTAGCTATTGTAATCCTCCGAGG
GGGCGAGCTCCCAAAAAAAAAAAAAAAAAAAAAAAAAAAAAA</td></tr>
<tr><td>FLuc-ms2L (polyA+)</td><td>T7 promoter
FLuc CDS
ms2L (4x)
3’ polyA (30A)</td><td>2C
2D
2E
S2
S3</td><td>TAATACGACTCACTATAGGGAGACCCAAGCTTATGCATGCGGCCGCATCTAGAGGGCCCGGATCCA
AATGGAAGACGCCAAAAACATAAAGAAAGGCCCGGCGCCATTCTATCCTCTAGAGGATGGAACCGC
TGGAGAGCAACTGCATAAGGCTATGAAGAGATACGCCCTGGTTCCTGGAACAATTGCTTTTACAGA
TGCACATATCGAGGTGAACATCACGTACGCGGAATACTTCGAAATGTCCGTTCGGTTGGCAGAAGC
TATGAAACGATATGGGCTGAATACAAATCACAGAATCGTCGTATGCAGTGAAAACTCTCTTCAATT
CTTTATGCCGGTGTTGGGCGCGTTATTTATCGGAGTTGCAGTTGCGCCCGCGAACGACATTTATAA
TGAACGTGAATTGCTCAACAGTATGAACATTTCGCAGCCTACCGTAGTGTTTGTTTCCAAAAAGGG
GTTGCAAAAAATTTTGAACGTGCAAAAAAAATTACCAATAATCCAGAAAATTATTATCATGGATTC
TAAAACGGATTACCAGGGATTTCAGTCGATGTACACGTTCGTCACATCTCATCTACCTCCCGGTTT
TAATGAATACGATTTTGTACCAGAGTCCTTTGATCGTGACAAAACAATTGCACTGATAATGAATTC
CTCTGGATCTACTGGGTTACCTAAGGGTGTGGCCCTTCCGCATAGAACTGCCTGCGTCAGATTCTC
GCATGCCAGAGATCCTATTTTTGGCAATCAAATCATTCCGGATACTGCGATTTTAAGTGTTGTTCC
ATTCCATCACGGTTTTGGAATGTTTACTACACTCGGATATTTGATATGTGGATTTCGAGTCGTCTT
AATGTATAGATTTGAAGAAGAGCTGTTTTTACGATCCCTTCAGGATTACAAAATTCAAAGTGCGTT
GCTAGTACCAACCCTATTTTCATTCTTCGCCAAAAGCACTCTGATTGACAAATACGATTTATCTAA
TTTACACGAAATTGCTTCTGGGGGCGCACCTCTTTCGAAAGAAGTCGGGGAAGCGGTTGCAAAACG
CTTCCATCTTCCAGGGATACGACAAGGATATGGGCTCACTGAGACTACATCAGCTATTCTGATTAC
ACCCGAGGGGGATGATAAACCGGGCGCGGTCGGTAAAGTTGTTCCATTTTTTGAAGCGAAGGTTGT
GGATCTGGATACCGGGAAAACGCTGGGCGTTAATCAGAGAGGCGAATTATGTGTCAGAGGACCTAT
GATTATGTCCGGTTATGTAAACAATCCGGAAGCGACCAACGCCTTGATTGACAAGGATGGATGGCT</td></tr>
</table>

			ACATTCTGGAGACATAGCTTACTGGGACGAAGACGAACACTTCTTCATAGTTGACCGCTTGAAGTC<br>TTTAATTAAATACAAAGGATATCAGGTGGCCCCCGCTGAATTGGAATCGATATTGTTACAACACCC<br>CAACATCTTCGACGCGGGCGTGGCAGGTCTTCCCGACGATGACGCCGGTGAACTTCCCGCCGCCGT<br>TGTTGTTTTGGAGCACGGAAAGACGATGACGGAAAAAGAGATCGTGGATTACGTCGCCAGTCAAGT<br>AACAACCGCGAAAAAGTTGCGCGGAGGAGTTGTGTTTGTGGACGAAGTACCGAAAGGTCTTACCGG<br>AAAACTCGACGCAAGAAAAATCAGAGAGATCCTCATAAAGGCCAAGAAGGGCGGAAAGTCCAAATT<br>GTAAAATGTAACTGTATTCAGCTCTCGGCATGGACGAGCTGTACAAGTAATTCTAGGCGATCGCTC<br>GAAAAACATGAGGATCACCCATGTCTGCAGGTCGACTCTAGAAAACATGAGGATCACCCATGTCCT<br>GCAGGTCGACTCTAGAAAACATGAGGATCACCCATGTCTGCAGGTCGACTCTAGAAAACATGAGGA<br>TCACCCATGTCCTCGAGTACAAGTAATTCTAGGCGATCGCTCGAAAAACATGAGGATCACCCATGT<br>CTGCAGGTCGACTCTAGAAAACATGAGGATCACCCATGTCCTGCAGGTCGACTCTAGAAAACATGA<br>GGATCACCCATGTCTGCAGGTCGACTCTAGAAAACATGAGGATCACCCATGTCCTCGAGGTGTGCG<br>GCCCTAGAGGGCCCGGTACCGTCGACGGATCCCTGCGATGCGAGACCGGTTAGCTATTGTAATCCT<br>CCGAGGGGGCGAGCTCCCAAAAAAAAAAAAAAAAAAAAAAAAAAAAAA
FLuc-ms2L (polyA-)	T7 promoter<br>FLuc CDS<br>ms2L (4x)	2C<br>S2	**TAATACGACTCACTATAGG**GAGACCCAAGCTTATGCATGCGGCCGCATCTAGAGGGCCCGGATCCA<br>AATGGAAGACGCCAAAAACATAAAGAAAGGCCCGGCGCCATTCTATCCTCTAGAGGATGGAACCGC<br>TGGAGAGCAACTGCATAAGGCTATGAAGAGATACGCCCTGGTTCCTGGAACAATTGCTTTTACAGA<br>TGCACATATCGAGGTGAACATCACGTACGCGGAATACTTCGAAATGTCCGTTCGGTTGGCAGAAGC<br>TATGAAACGATATGGGCTGAATACAAATCACAGAATCGTCGTATGCAGTGAAAACTCTCTTCAATT<br>CTTTATGCCGGTGTTGGGCGCGTTATTTATCGGAGTTGCAGTTGCGCCCGCGAACGACATTTATAA<br>TGAACGTGAATTGCTCAACAGTATGAACATTTCGCAGCCTACCGTAGTGTTTGTTTCCAAAAAGGG<br>GTTGCAAAAAATTTTGAACGTGCAAAAAAAATTACCAATAATCCAGAAAATTATTATCATGGATTC<br>TAAAACGGATTACCAGGGATTTCAGTCGATGTACACGTTCGTCACATCTCATCTACCTCCCGGTTT<br>TAATGAATACGATTTTGTACCAGAGTCCTTTGATCGTGACAAAACAATTGCACTGATAATGAATTC<br>CTCTGGATCTACTGGGTTACCTAAGGGTGTGGCCCTTCCGCATAGAACTGCCTGCGTCAGATTCTC<br>GCATGCCAGAGATCCTATTTTTGGCAATCAAATCATTCCGGATACTGCGATTTTAAGTGTTGTTCC<br>ATTCCATCACGGTTTTGGAATGTTTACTACACTCGGATATTTGATATGTGGATTTCGAGTCGTCTT<br>AATGTATAGATTTGAAGAAGAGCTGTTTTTACGATCCCTTCAGGATTACAAAATTCAAAGTGCGTT<br>GCTAGTACCAACCCTATTTTCATTCTTCGCCAAAAGCACTCTGATTGACAAATACGATTTATCTAA<br>TTTACACGAAATTGCTTCTGGGGGCGCACCTCTTTCGAAAGAAGTCGGGGAAGCGGTTGCAAAACG<br>CTTCCATCTTCCAGGGATACGACAAGGATATGGGCTCACTGAGACTACATCAGCTATTCTGATTAC<br>ACCCGAGGGGGATGATAAACCGGGCGCGGTCGGTAAAGTTGTTCCATTTTTTGAAGCGAAGGTTGT<br>GGATCTGGATACCGGGAAAACGCTGGGCGTTAATCAGAGAGGCGAATTATGTGTCAGAGGACCTAT<br>GATTATGTCCGGTTATGTAAACAATCCGGAAGCGACCAACGCCTTGATTGACAAGGATGGATGGCT<br>ACATTCTGGAGACATAGCTTACTGGGACGAAGACGAACACTTCTTCATAGTTGACCGCTTGAAGTC<br>TTTAATTAAATACAAAGGATATCAGGTGGCCCCCGCTGAATTGGAATCGATATTGTTACAACACCC<br>CAACATCTTCGACGCGGGCGTGGCAGGTCTTCCCGACGATGACGCCGGTGAACTTCCCGCCGCCGT<br>TGTTGTTTTGGAGCACGGAAAGACGATGACGGAAAAAGAGATCGTGGATTACGTCGCCAGTCAAGT<br>AACAACCGCGAAAAAGTTGCGCGGAGGAGTTGTGTTTGTGGACGAAGTACCGAAAGGTCTTACCGG<br>AAAACTCGACGCAAGAAAAATCAGAGAGATCCTCATAAAGGCCAAGAAGGGCGGAAAGTCCAAATT<br>GTAAAATGTAACTGTATTCAGCTCTCGGCATGGACGAGCTGTACAAGTAATTCTAGGCGATCGCTC<br>GAAAAACATGAGGATCACCCATGTCTGCAGGTCGACTCTAGAAAACATGAGGATCACCCATGTCCT<br>GCAGGTCGACTCTAGAAAACATGAGGATCACCCATGTCTGCAGGTCGACTCTAGAAAACATGAGGA<br>TCACCCATGTCCTCGAGTACAAGTAATTCTAGGCGATCGCTCGAAAAACATGAGGATCACCCATGT<br>CTGCAGGTCGACTCTAGAAAACATGAGGATCACCCATGTCCTGCAGGTCGACTCTAGAAAACATGA<br>GGATCACCCATGTCTGCAGGTCGACTCTAGAAAACATGAGGATCACCCATGTCCTCGAGGTGTGCG<br>GCCCTAGAGGGCCCGGTA
MS2-CNOT7	T7 promoter<br>MS2-CNOT7 CDS<br>3' polyA (30A)	2C<br>2D<br>2E<br>S2<br>S3	**TAATACGACTCACTATAGG**GAGACCCAAGCTTATGCATGCGGCCCCGCCACCATGGCTTCTAACTT<br>TACTCAGTTCGTTCTCGTCGACAATGGCGGAACTGGCGACGTGACTGTCGCCCCAAGCAACTTCGC<br>TAACGGGGTCGCTGAATGGATCAGCTCTAACTCGCGTTCACAGGCTTACAAAGTAACCTGTAGCGT<br>TCGTCAGAGCTCTGCGCAGAAGCGCAAATACACCATCAAAGTCGAGGTGCCTAAAGTGGCAACCCA<br>GACTGTTGGTGGTGTAGAGCTTCCTGTAGCCGCATGGCGTTCGTACTTAAATATGGAACTAACCAT<br>TCCAATTTTCGCCACGAATTCCGACTGCGAGCTTATTGTTAAGGCAATGCAAGGTCTCCTAAAAGA<br>TGGAAACCCGATTCCCTCGGCCATCGCAGCAAACTCCGGCATCTACAAGCTTAATAATGGTACCAT<br>GCCAGCGGCAACTGTAGATCATAGCCAAAGAATTTGTGAAGTTTGGGCTTGCAACTTGGATGAAGA<br>GATGAAGAAAATTCGTCAAGTTATCCGAAAATATAATTACGTTGCTATGGACACCGAGTTTCCAGG<br>TGTGGTTGCAAGACCCATTGGAGAATTCAGGAGCAATGCTGACTATCAATACCAACTATTGCGGTG<br>TAATGTAGACTTGTTAAAGATAATTCAGCTAGGACTGACATTTATGAATGAGCAAGGAGAATACCC<br>TCCAGGAACTTCAACTTGGCAGTTTAATTTTAAATTTAATTTGACGGAGGACATGTATGCCCAGGA<br>CTCTATAGAGCTACTAACAACATCTGGTATCCAGTTTAAAAAACATGAGGAGGAAGGAATTGAAAC<br>CCAGTACTTTGCAGAACTTCTTATGACTTCTGGAGTGGTCCTCTGTGAAGGGGTCAAATGGTTGTC<br>ATTTCATAGCGGTTACGACTTTGGCTACTTAATCAAAATCCTAACCAACTCTAACTTGCCTGAAGA<br>AGAACTTGACTTCTTTGAGATCCTTCGATTGTTTTTCCTGTCATTTATGATGTGAAGTACCTCAT<br>GAAGAGCTGCAAAAATCTCAAAGGTGGATTACAGGAGGTGGCAGAACAGTTAGAGCTGGAACGGAT<br>AGGACCACAACATCAGGCAGGATCTGATTCATTGCTCACAGGAATGGCCTTTTTCAAAATGAGAGA<br>AATGTTCTTTGAAGATCATATTGATGATGCCAAATATTGTGGTCATTTGTATGGCCTTGGTTCTGG<br>TTCATCCTATGTACAGAATGGCACAGGGAATGCATATGAAGAGGAAGCCAACAAGCAGTCAGTTTA<br>ACTAGAGGGCCCGGTACCGTCGACGGATCCCTGCGATGCGAGACCGGTTAGCTATTGTAATCCTCC<br>GAGGGGGCGAGCTCCCAAAAAAAAAAAAAAAAAAAAAAAAAAAAAA
MS2*-CNOT7	T7 promoter<br>MS2-CNOT7 CDS	2C<br>2D	**TAATACGACTCACTATAGG**GAGACCCAAGCTCATGGCTTCTAACTTTACTCAGTTCGTTCTCGTCG<br>ACAATGGCGGAACTGGCGACGTGACTGTCGCCCCAAGCAACTTCGCTAACGGGGTCGCTGAATGGA

	3’ polyA (30A)<br>**AAA**: K56D and K58E mutations	S2<br>S3	TCAGCTCTAACTCGCGTTCACAGGCTTACAAAGTAACCTGTAGCGTTCGTCAGAGCTCTGCGCAG**G**<br>**AC**CGC**GAA**TACACCATCAAAGTCGAGGTGCCTAAAGTGGCAACCCAGACTGTTGGTGGTGTAGAGC<br>TTCCTGTAGCCGCATGGCGTTCGTACTTAAATATGGAACTAACCATTCCAATTTTCGCCACGAATT<br>CCGACTGCGAGCTTATTGTTAAGGCAATGCAAGGTCTCCTAAAAGATGGAAACCCGATTCCCTCGG<br>CCATCGCAGCAAACTCCGGCATCTACAAGCTTAATAATGGTACCATGCCAGCGGCAACTGTAGATC<br>ATAGCCAAAGAATTTGTGAAGTTTGGGCTTGCAACTTGGATGAAGAGATGAAGAAAATTCGTCAAG<br>TTATCCGAAAATATAATTACGTTGCTATGGACACCGAGTTTCCAGGTGTGGTTGCAAGACCCATTG<br>GAGAATTCAGGAGCAATGCTGACTATCAATACCAACTATTGCGGTGTAATGTAGACTTGTTAAAGA<br>TAATTCAGCTAGGACTGACATTTATGAATGAGCAAGGAGAATACCCTCCAGGAACTTCAACTTGGC<br>AGTTTAATTTTAAATTTAATTTGACGGAGGACATGTATGCCCAGGACTCTATAGAGCTACTAACAA<br>CATCTGGTATCCAGTTTAAAAAACATGAGGAGGAAGGAATTGAAACCCAGTACTTTGCAGAACTTC<br>TTATGACTTCTGGAGTGGTCCTCTGTGAAGGGGTCAAATGGTTGTCATTTCATAGCGGTTACGACT<br>TTGGCTACTTAATCAAAATCCTAACCAACTCTAACTTGCCTGAAGAAGAACTTGACTTCTTTGAGA<br>TCCTTCGATTGTTTTTTCCTGTCATTTATGATGTGAAGTACCTCATGAAGAGCTGCAAAAATCTCA<br>AAGGTGGATTACAGGAGGTGGCAGAACAGTTAGAGCTGGAACGGATAGGACCACAACATCAGGCAG<br>GATCTGATTCATTGCTCACAGGAATGGCCTTTTTCAAAATGAGAGAAATGTTCTTTGAAGATCATA<br>TTGATGATGCCAAATATTGTGGTCATTTGTATGGCCTTGGTTCTGGTTCATCCTATGTACAGAATG<br>GCACAGGGAATGCATATGAAGAGGAAGCCAACAAGCAGTCAGTTTAAGATCCCTGCGATGCGAGAC<br>CGGTTAGCTATTGTAATCCTCCGAGGGGGCGAGCTCCCAAAAAAAAAAAAAAAAAAAAAAAAAAAA<br>AA
MS2	T7 promoter<br>MS2 CDS<br>3’ polyA (30A)	2D<br>2E	**TAATACGACTCACTATAGG**GAGACCCAAGCTTATGCATGCGGCCCCGCCACCATGGCTTCTAACTT<br>TACTCAGTTCGTTCTCGTCGACAATGGCGGAACTGGCGACGTGACTGTCGCCCCAAGCAACTTCGC<br>TAACGGGGTCGCTGAATGGATCAGCTCTAACTCGCGTTCACAGGCTTACAAAGTAACCTGTAGCGT<br>TCGTCAGAGCTCTGCGCAGAAGCGCAAATACACCATCAAAGTCGAGGTGCCTAAAGTGGCAACCCA<br>GACTGTTGGTGGTGTAGAGCTTCCTGTAGCCGCATGGCGTTCGTACTTAAATATGGAACTAACCAT<br>TCCAATTTTCGCCACGAATTCCGACTGCGAGCTTATTGTTAAGGCAATGCAAGGTCTCCTAAAAGA<br>TGGAAACCCGATTCCCTCGGCCATCGCAGCAAACTCCGGCATCTACAAGCTTAATAATGGTACAAA<br>AAAAAAAAAAAAAAAAAAAAAAAAAAA
MS2*	T7 promoter<br>MS2* CDS<br>3’ polyA (30A)	2D	**TAATACGACTCACTATAGG**GAGACCCAAGCTTATGCATGCGGCCCCGCCACCATGGCTTCTAACTT<br>TACTCAGTTCGTTCTCGTCGACAATGGCGGAACTGGCGACGTGACTGTCGCCCCAAGCAACTTCGC<br>TAACGGGGTCGCTGAATGGATCAGCTCTAACTCGCGTTCACAGGCTTACAAAGTAACCTGTAGCGT<br>TCGTCAGAGCTCTGCGCAG**GAC**CGC**GAA**TACACCATCAAAGTCGAGGTGCCTAAAGTGGCAACCCA<br>GACTGTTGGTGGTGTAGAGCTTCCTGTAGCCGCATGGCGTTCGTACTTAAATATGGAACTAACCAT<br>TCCAATTTTCGCCACGAATTCCGACTGCGAGCTTATTGTTAAGGCAATGCAAGGTCTCCTAAAAGA<br>TGGAAACCCGATTCCCTCGGCCATCGCAGCAAACTCCGGCATCTACAAGCTTAATAATGGTACAAA<br>AAAAAAAAAAAAAAAAAAAAAAAAAAA
FLuc (ms2L-)	T7 promoter<br>FLuc CDS<br>3’ polyA (30A)	2E	**TAATACGACTCACTATAGG**GAATACAAGCTTATGCATGCGGCCGCATCTAGAGGGCCCGGATCCAA<br>ATGGAAGACGCCAAAAACATAAAGAAAGGCCCGGCGCCATTCTATCCTCTAGAGGATGGAACCGCT<br>GGAGAGCAACTGCATAAGGCTATGAAGAGATACGCCCTGGTTCCTGGAACAATTGCTTTTACAGAT<br>GCACATATCGAGGTGAACATCACGTACGCGGAATACTTCGAAATGTCCGTTCGGTTGGCAGAAGCT<br>ATGAAACGATATGGGCTGAATACAAATCACAGAATCGTCGTATGCAGTGAAAACTCTCTTCAATTC<br>TTTATGCCGGTGTTGGGCGCGTTATTTATCGGAGTTGCAGTTGCGCCCGCGAACGACATTTATAAT<br>GAACGTGAATTGCTCAACAGTATGAACATTTCGCAGCCTACCGTAGTGTTTGTTTCCAAAAAGGGG<br>TTGCAAAAAATTTTGAACGTGCAAAAAAAATTACCAATAATCCAGAAAATTATTATCATGGATTCT<br>AAAACGGATTACCAGGGATTTCAGTCGATGTACACGTTCGTCACATCTCATCTACCTCCCGGTTTT<br>AATGAATACGATTTTGTACCAGAGTCCTTTGATCGTGACAAAACAATTGCACTGATAATGAATTCC<br>TCTGGATCTACTGGGTTACCTAAGGGTGTGGCCCTTCCGCATAGAACTGCCTGCGTCAGATTCTCG<br>CATGCCAGAGATCCTATTTTTGGCAATCAAATCATTCCGGATACTGCGATTTTAAGTGTTGTTCCA<br>TTCCATCACGGTTTTGGAATGTTTACTACACTCGGATATTTGATATGTGGATTTCGAGTCGTCTTA<br>ATGTATAGATTTGAAGAAGAGCTGTTTTTACGATCCCTTCAGGATTACAAAATTCAAAGTGCGTTG<br>CTAGTACCAACCCTATTTTCATTCTTCGCCAAAAGCACTCTGATTGACAAATACGATTTATCTAAT<br>TTACACGAAATTGCTTCTGGGGGCGCACCTCTTTCGAAAGAAGTCGGGGAAGCGGTTGCAAAACGC<br>TTCCATCTTCCAGGGATACGACAAGGATATGGGCTCACTGAGACTACATCAGCTATTCTGATTACA<br>CCCGAGGGGGATGATAAACCGGGCGCGGTCGGTAAAGTTGTTCCATTTTTTGAAGCGAAGGTTGTG<br>GATCTGGATACCGGGAAAACGCTGGGCGTTAATCAGAGAGGCGAATTATGTGTCAGAGGACCTATG<br>ATTATGTCCGGTTATGTAAACAATCCGGAAGCGACCAACGCCTTGATTGACAAGGATGGATGGCTA<br>CATTCTGGAGACATAGCTTACTGGGACGAAGACGAACACTTCTTCATAGTTGACCGCTTGAAGTCT<br>TTAATTAAATACAAAGGATATCAGGTGGCCCCCGCTGAATTGGAATCGATATTGTTACAACACCCC<br>AACATCTTCGACGCGGGCGTGGCAGGTCTTCCCGACGATGACGCCGGTGAACTTCCCGCCGCCGTT<br>GTTGTTTTGGAGCACGGAAAGACGATGACGGAAAAAGAGATCGTGGATTACGTCGCCAGTCAAGTA<br>ACAACCGCGAAAAAGTTGCGCGGAGGAGTTGTGTTTGTGGACGAAGTACCGAAAGGTCTTACCGGA<br>AAACTCGACGCAAGAAAAATCAGAGAGATCCTCATAAAGGCCAAGAAGGGCGGAAAGTCCAAATTG<br>TAAAATGTAACTGTATTCAGCGATGACGAAATTCTTAGCTATTGTAATCCTCCGAGGGGGCGAGCT<br>CCCAAAAAAAAAAAAAAAAAAAAAAAAAAAAAACCGAATT
K-turn-NLuc	T7 promoter<br>ATG start codon<br>K-turn (pboxC/D)<br>AAA linker<br>NLuc CDS<br>3’ polyA (30A)	3C<br>S4A	**TAATACGACTCACTATAGG**GAGACCCAAGCTTCGCCACCATGGGCGTGATGCGAAAGCTGACCCT<br>GTGAGCAAGGGCGAGGAGCTGTTCACCGTCTTCACACTCGAAGATTTCGTTGGGGACTGGCGACAG<br>ACAGCCGGCTACAACCTGGACCAAGTCCTTGAACAGGGAGGTGTGTCCAGTTTGTTTCAGAATCTC<br>GGGGTGTCCGTAACTCCGATCCAAAGGATTGTCCTGAGCGGTGAAAATGGGCTGAAGATCGACATC<br>CATGTCATCATCCCGTATGAAGGTCTGAGCGGCGACCAAATGGGCCAGATCGAAAAAATTTTTAAG<br>GTGGTGTACCCTGTGGATGATCATCACTTTAAGGTGATCCTGCACTATGGCACACTGGTAATCGAC<br>GGGGTTACGCCGAACATGATCGACTATTTCGGACGGCCGTATGAAGGCATCGCCGTGTTCGACGGC<br>AAAAAGATCACTGTAACAGGGACCCTGTGGAACGGCAACAAAATTATCGACGAGCGCCTGATCAAC

			CCCGACGGCTCCCTGCTGTTCCGAGTAACCATCAACGGAGTGACCGGCTGGCGGCTGTGCGAACGCATTCTGGCGTAAGATCCCTGCGATGCGAGACCGGTTAGCTATTGTAATCCTCCGAGGGGGCGAGCTCCCAAAAAAAAAAAAAAAAAAAAAAAAAAAAAA
K-turn-NLuc*	**T7 promoter**<br><u>ATG</u> start codon<br><u>K-turn*</u><br><u>(p1boxC/D*)</u><br>AAA linker<br>NLuc CDS<br>3’ polyA (30A)	3C<br>S4A	**TAATACGACTCACTATAGG**GAGACCCAAGCTGCCACC<u>ATG</u><u>GGGCGTCATCCGAAAGGTGCCCCG</u>GTGAGCAAGGGCGAGGAGCTGTTCACCGTCTTCACACTCGAAGATTTCGTTGGGGACTGGCGACAGACAGCCGGCTACAACCTGGACCAAGTCCTTGAACAGGGAGGTGTGTCCAGTTTGTTTCAGAATCTCGGGGTGTCCGTAACTCCGATCCAAAGGATTGTCCTGAGCGGTGAAAATGGGCTGAAGATCGACATCCATGTCATCATCCCGTATGAAGGTCTGAGCGGCGACCAAATGGGCCAGATCGAAAAAATTTTTAAGGTGGTGTACCCTGTGGATGATCATCACTTTAAGGTGATCCTGCACTATGGCACACTGGTAATCGACGGGGTTACGCCGAACATGATCGACTATTTCGGACGGCCGTATGAAGGCATCGCCGTGTTCGACGGCAAAAAGATCACTGTAACAGGGACCCTGTGGAACGGCAACAAAATTATCGACGAGCGCCTGATCAACCCCGACGGCTCCCTGCTGTTCCGAGTAACCATCAACGGAGTGACCGGCTGGCGGCTGTGCGAACGCATTCTGGCGTAACTAGAGGGCCCGGTACCGTCGACGGATCCCTGCGATGCGAGACCGGTTAGCTATTGTAATCCTCCGAGGGGGCGAGCTCCCAAAAAAAAAAAAAAAAAAAAAAAAAAAAAA
L7Ae	**T7 promoter**<br>L7Ae CDS<br>3’ polyA (30A)	3C	**TAATACGACTCACTATAGG**GAGACCCAAGCTGCCACCATGCATCATCACCATCATCACTACGTGAGATTTGAGGTTCCTGAGGACATGCAGAACGAAGCTCTGAGTCTGCTGGAGAAGGTTAGGGAGAGCGGTAAGGTAAAGAAAGGTACCAACGAGACGACAAAGGCTGTGGAGAGGGGACTGGCAAAGCTCGTTTACATCGCAGAGGATGTTGACCCGCCTGAGATCGTTGCTCATCTGCCCCTCCTCTGCGAGGAGAAGAATGTGCCGTACATTTACGTTAAAAGCAAGAACGACCTTGGAAGGGCTGTGGGCATTGAGGTGCCATGCGCTTCGGCAGCGATAATCAACGAGGGAGAGCTGAGAAAGGAGCTTGGAAGCCTTGTGGAGAAGATTAAAGGCCTTCAGAAGTAATAGAGGGCCCGGTACCGTCGACGGATCCCTGCGATGCGAGACCGGTTAGCTATTGTAATCCTCCGAGGGGGCGAGCTCCCAAAAAAAAAAAAAAAAAAAAAAAAAAAAAA
L7Ae(TCS)	**T7 promoter**<br>L7Ae(**TCS**) CDS<br>3’ polyA (30A)	3C	**TAATACGACTCACTATAGG**GAGACCCAAGCTGCCACCATGCATCATCACCATCATCACGGTTACGTGAGATTTGAGGTTCCTGAGGACATGCAGAACGAAGCTCTGAGTCTGCTGGAGAAGGTTAGGGAGAGCGGTAAGGTAAAGAAAGGTACCAACGAGACGACAAAGGCTGTGGAGAGGGGACTGGCAAAGCTCGTTTACATCGCAGAGGATGTTGACCCGCCTGAGATCGTTGCTCATCTGCCCCTCCTCTGCGAGGAGAAGAATGTGCCGTACATTTACGTTAAA**GAAAACCTGTACTTCCAGTCC**AGCAAGAACGACCTTGGAAGGGCTGTGGGCATTGAGGTGCCATGCGCTTCGGCAGCGATAATCAACGAGGGAGAGCTGAGAAAGGAGCTTGGAAGCCTTGTGGAGAAGATTAAAGGCCTTCAGAAGTAACTAGAGGGCCCGGTACCGTCGACGGATCCCTGCGATGCGAGACCGGTTAGCTATTGTAATCCTCCGAGGGGGCGAGCTCCCAAAAAAAAAAAAAAAAAAAAAAAAAAAAAA

Table S2. DNA Insert sequences in plasmids used for TX-TL reactions.

Insert name	Platform	Backbone	Used in Fig	Elements	Insert sequence (nt)
K-turn-NLuc	*E. coli* S30	BB1	3E<br>S4B	K-turn (pboxC/D)<br>AAA linker<br>NLuc CDS	GGATGGGCGTGATGCGAAAGCTGACCCTGTGAGCAAGGGCGAGGAGCTGTTCACCGTCT<br>TCACACTCGAAGATTTCGTTGGGGACTGGCGCCAGACAGCCGGCTACAACCTGGACCAA<br>GTCCTTGAACAGGGAGGTGTGTCCAGTTTGTTTCAGAATCTCGGGGTGTCCGTAACTCC<br>GATCCAACGCATTGTCCTGAGCGGTGAAAATGGGCTGAAGATCGACATCCATGTCATCA<br>TCCCGTATGAAGGTCTGAGCGGCGACCAAATGGGCCAGATCGAAAAAATTTTTAAGGTG<br>GTGTACCCTGTGGATGATCATCACTTTAAGGTGATCCTGCACTATGGCACACTGGTAAT<br>CGACGGGGTTACGCCGAACATGATCGACTATTTCGGACGGCCGTATGAAGGCATCGCCG<br>TGTTCGACGGCAAAAAGATCACTGTAACAGGGACCCTGTGGAACGGCAACAAAATTATC<br>GACGAGCGCCTGATCAACCCCGACGGCTCCCTGCTGTTCCGCGTAACCATCAACGGAGT<br>GACCGGCTGGCGGCTGTGCGAACGCATTCTGGCGTAACTCGAGCGGTACCGT
K-turn-NLuc*	*E. coli* S30	BB1	3E<br>S4B	K-turn* (p1boxC/D*)<br>AAA linker<br>NLuc CDS	GGATGGGCGTCATCCGAAAGGTGCCCCGGTGAGCAAGGGCGAGGAGCTGTTCACCGTCT<br>TCACACTCGAAGATTTCGTTGGGGACTGGCGCCAGACAGCCGGCTACAACCTGGACCAA<br>GTCCTTGAACAGGGAGGTGTGTCCAGTTTGTTTCAGAATCTCGGGGTGTCCGTAACTCC<br>GATCCAACGCATTGTCCTGAGCGGTGAAAATGGGCTGAAGATCGACATCCATGTCATCA<br>TCCCGTATGAAGGTCTGAGCGGCGACCAAATGGGCCAGATCGAAAAAATTTTTAAGGTG<br>GTGTACCCTGTGGATGATCATCACTTTAAGGTGATCCTGCACTATGGCACACTGGTAAT<br>CGACGGGGTTACGCCGAACATGATCGACTATTTCGGACGGCCGTATGAAGGCATCGCCG<br>TGTTCGACGGCAAAAAGATCACTGTAACAGGGACCCTGTGGAACGGCAACAAAATTATC<br>GACGAGCGCCTGATCAACCCCGACGGCTCCCTGCTGTTCCGCGTAACCATCAACGGAGT<br>GACCGGCTGGCGGCTGTGCGAACGCATTCTGGCGTAACTCGAGCGGTACCGTGAATTCA<br>AGGGCGA
L7Ae	*E. coli* S30<br>PURE	BB1/BB2	3E (BB1)<br>3I-J<br>4C-E<br>4G-I<br>5A<br>S7-S9	L7Ae CDS	TACGTGCGCTTTGAGGTTCCTGAGGACATGCAGAACGAAGCTCTGAGTCTGCTGGAGAA<br>GGTTCGCGAGAGCGGTAAGGTAAAGAAAGGTACCAACGAGACGACAAAGGCTGTGGAGC<br>GCGGACTGGCAAAGCTCGTTTACATCGCAGAGGATGTTGACCCGCCTGAGATCGTTGCT<br>CATCTGCCCCTCCTCTGCGAGGAGAAGAATGTGCCGTACATTTACGTTAAAAGCAAGAA<br>CGACCTTGGACGCGCTGTGGGCATTGAGGTGCCATGCGCTTCGGCAGCGATTATCAACG<br>AGGGAGAGCTGCGCAAGGAGCTTGGAAGCCTTGTGGAGAAGATTAAAGGCCTTCAGAAG<br>TAA
L7Ae*	*E. coli* S30<br>PURE	BB1/BB2	3I-J<br>4D-E<br>4H-I<br>S7-S9	L7Ae* CDS	TACGTGCGCTTTGAGGTTCCTGAGGACATGCAGAACGAAGCTCTGAGTCTGCTGGAGAA<br>GGTTCGCGAGAGCGGTAAGGTAAAGAAAGGTACCAACGAGACGACAGCGGCTGTGGAGC<br>GCGGACTGGCAAAGCTCGTTTACATCGCAGAGGATGTTGACCCGCCTGAGATCGTTGCT<br>CATCTGCCCCTCCTCTGCGAGGAGAAGAATGTGCCGTACATTTACGTTAAAAGCGCGAA<br>CGACCTTGGACGCGCTGTGGGCATTGAGGTGCCATGCGCTTCGGCAGCGATTATCAACG<br>AGGGAGAGCTGCGCAAGGAGCTTGGAAGCCTTGTGGAGAAGATTAAAGGCCTTCAGAAG<br>TAA
K-turn-EGFP	*E. coli* S30<br>PURE	BB1/BB2	3I-J<br>4C-E<br>4G-I<br>5A<br>5D-F<br>S6-S10	K-turn (pboxC/D)<br>EGFP (CDS)	ATTATGGGGCGTGATGCGAAAGCTGACCCTGTGAGCAAGGGCGAGGAGCTGTTCACCGG<br>GGTGGTGCCCATCCTGGTCGAGCTGGACGGCGACGTAAACGGCCACAAGTTCAGCGTGT<br>CCGGCGAGGGCGAGGGCGATGCCACCTACGGCAAGCTGACCCTGAAGTTCATCTGCACC<br>ACCGGCAAGCTGCCCGTGCCCTGGCCCACCCTCGTGACCACCCTGACCTACGGCGTGCA<br>GTGCTTCAGCCGCTACCCCGACCACATGAAGCAGCACGACTTCTTCAAGTCCGCCATGC<br>CCGAAGGCTACGTCCAGGAGCGCACCATCTTCTTCAAGGACGACGGCAACTACAAGACC<br>CGCGCCGAGGTGAAGTTCGAGGGCGACACCCTGGTGAACCGCATCGAGCTGAAGGGCAT<br>CGACTTCAAGGAGGACGGCAACATCCTGGGGCACAAGCTGGAGTACAACTACAACAGCC<br>ACAACGTCTATATCATGGCCGACAAGCAGAAGAACGGCATCAAGGTGAACTTCAAGATC<br>CGCCACAACATCGAGGACGGCAGCGTGCAGCTCGCCGACCACTACCAGCAGAACACCCC<br>CATCGGCGACGGCCCCGTGCTGCTGCCCGACAACCACTACCTGAGCACCCAGTCCGCCC<br>TGAGCAAAGACCCCAACGAGAAGCGCGATCACATGGTCCTGCTGGAGTTCGTGACCGCC<br>GCCGGGATCACTCTCGGCATGGACGAGCTGTACAAGTAA
K-turn-EGFP*	*E. coli* S30<br>PURE	BB1/BB2	3I-J<br>4C<br>4G<br>4I<br>5A<br>5D-F<br>S6-S7<br>S9-S10	K-turn* (pboxC/D*)<br>EGFP (CDS)	GGGCGTCATGCGAAAGCTGCCCCGGTGAGCAAGGGCGAGGAGCTGTTCACCGGGGTGGT<br>GCCCATCCTGGTCGAGCTGGACGGCGACGTAAACGGCCACAAGTTCAGCGTGTCCGGCG<br>AGGGCGAGGGCGATGCCACCTACGGCAAGCTGACCCTGAAGTTCATCTGCACCACCGGC<br>AAGCTGCCCGTGCCCTGGCCCACCCTCGTGACCACCCTGACCTACGGCGTGCAGTGCTT<br>CAGCCGCTACCCCGACCACATGAAGCAGCACGACTTCTTCAAGTCCGCCATGCCCGAAG<br>GCTACGTCCAGGAGCGCACCATCTTCTTCAAGGACGACGGCAACTACAAGACCCGCGCC<br>GAGGTGAAGTTCGAGGGCGACACCCTGGTGAACCGCATCGAGCTGAAGGGCATCGACTT<br>CAAGGAGGACGGCAACATCCTGGGGCACAAGCTGGAGTACAACTACAACAGCCACAACG<br>TCTATATCATGGCCGACAAGCAGAAGAACGGCATCAAGGTGAACTTCAAGATCCGCCAC<br>AACATCGAGGACGGCAGCGTGCAGCTCGCCGACCACTACCAGCAGAACACCCCCATCGG<br>CGACGGCCCCGTGCTGCTGCCCGACAACCACTACCTGAGCACCCAGTCCGCCCTGAGCA<br>AAGACCCCAACGAGAAGCGCGATCACATGGTCCTGCTGGAGTTCGTGACCGCCGCCGGG<br>ATCACTCTCGGCATGGACGAGCTGTACAAGTAA
L7Ae(TCS)	PURE	BB2	5A<br>5D-F<br>S10	L7Ae(TCS) CDS	ACGTGCGCTTTGAGGTTCCTGAGGACATGCAGAACGAAGCTCTGAGTCTGCTGGAGAAG<br>GTTCGCGAGAGCGGTAAGGTAAAGAAAGGTACCAACGAGACGACAAAGGCTGTGGAGCG<br>CGGACTGGCAAAGCTCGTTTACATCGCAGAGGATGTTGACCCGCCTGAGATCGTTGCTC<br>ATCTGCCCCTCCTCTGCGAGGAGAAGAATGTGCCGTACATTTACGTTAAA**GAAAACCTG**<br>**TACTTCCAGTCC**AGCAAGAACGACCTTGGACGCGCTGTGGGCATTGAGGTGCCATGCGC<br>TTCGGCAGCGATTATCAACGAGGGAGAGCTGCGCAAGGAGCTTGGAAGCCTTGTGGAGA<br>AGATTAAAGGCCTTCAGAAGTAA

Table S3. Oligos and primers used in this study.

Name	Sequence (5’-3’)	Application	Type/Modification
T7p_Fw	GAAATTAATACGACTCACTATAGGGAGACCCAAGCT	IVT template generation (PCR) (all templates)	DNA oligo, unmodified
PolyA+_Rev	TTTTTTTTTTTTTTTTTTTTTTTTTTTTTTGGGAGCTCGCCCCCTCGGAG	IVT template generation (PCR) (polyA+ templates)	DNA oligo, unmodified
PolyA-_Rev	TTGGGAGCTCGCCCCCT	IVT template generation (PCR) (polyA- templates)	DNA oligo, unmodified
MS2-PolyA+_Rev	TTTTTTTTTTTTTTTTTTTTTTTTTTTTTTGTACCATTATTAAGCT	IVT template generation (PCR) (MS2 and MS2* constructs)	DNA oligo, unmodified
miR-451-TS-S1	CTAGGGTACCAACTCAGTAATGGTAACGGTTTAACTCAGTAATGGTAACG	Oligo annealing (Sense #1) for miR-451 TS generation	DNA oligo, unmodified
miR-451-TS-AS1	AAACCGTTACCATTACTGAGTTAAACCGTTACCATTACTGAGTTGGTACC	Oligo annealing (Antisense #1) for miR-451 TS generation	DNA oligo, unmodified
miR-451-TS-S2	GTTTAACTCAGTAATGGTAACGGTTTAACTCAGTAATGGTAACGGTTTCCCGGG	Oligo annealing (Sense #2) for miR-451 TS generation	DNA oligo, unmodified
miR-451-TS-AS2	TCGACCCGGGAAACCGTTACCATTACTGAGTTAAACCGTTACCATTACTGAGTT	Oligo annealing (Antisense #2) for miR-451 TS generation	DNA oligo, unmodified
let-7-TS-S1	CTAGGGTACCAACTATACAACCTACTACCTCAAACTATACAACCTACTAC	Oligo annealing (Sense #1) for let-7 TS generation	DNA oligo, unmodified
let-7-TS-AS1	TGAGGTAGTAGGTTGTATAGTTTGAGGTAGTAGGTTGTATAGTTGGTACC	Oligo annealing (Antisense #1) for let-7 TS generation	DNA oligo, unmodified
let-7-TS-S2	CTCAAACTATACAACCTACTACCTCAAACTATACAACCTACTACCTCACCCGGG	Oligo annealing (Sense #2) for let-7 TS generation	DNA oligo, unmodified
let-7-TS-AS2	TCGACCCGGGTGAGGTAGTAGGTTGTATAGTTTGAGGTAGTAGGTTGTATAGTT	Oligo annealing (Antisense #2) for let-7 TS generation	DNA oligo, unmodified
miR-221-TS-S1	CTAGGGTACCGAAACCCAGCAGACAATGTAGCTGAAACCCAGCAGACAATGT	Oligo annealing (Sense #1) for miR-221 TS generation	DNA oligo, unmodified
miR-221-TS-AS1	AGCTACATTGTCTGCTGGGTTTCAGCTACATTGTCTGCTGGGTTTCGGTACC	Oligo annealing (Antisense #1) for miR-221 TS generation	DNA oligo, unmodified
miR-221-TS-S2	AGCTGAAACCCAGCAGACAATGTAGCTGAAACCCAGCAGACAATGTAGCTCCCGGG	Oligo annealing (Sense #2) for miR-221 TS generation	DNA oligo, unmodified
miR-221-TS-AS2	TCGACCCGGGAGCTACATTGTCTGCTGGGTTTCAGCTACATTGTCTGCTGGGTTTC	Oligo annealing (Antisense #2) for miR-221 TS generation	DNA oligo, unmodified
Anti-miR-451_2OMe	AACUCAGUAAUGGUAACGGUUU	Anti-miRNA (miR-451)	RNA oligo, 2’ O-Me modified bases
Anti-let-7_2OMe	AACUAUACAACCUACUACCUCA	Anti-miRNA (let-7)	RNA oligo, 2’ O-Me modified bases
Anti-miR-221_2OMe	GAAACCCAGCAGACAAUGUAGCU	Anti-miRNA (miR-221)	RNA oligo, 2’ O-Me modified bases
Nluc2	AGGGUCCCUGUUACAGUGAUC	Synthetic miRNA (qPCR standard)	5’-Phosphorylated RNA oligo
miR-451a_qPCR_Fw	AAACCGTTACCATTACTGAGTT	miR-451 specific F primer (qPCR)	DNA oligo, unmodified
Let-7a_qPCR_Fw	TGAGGTAGTAGGTTGTATAGTT	Let-7 specific F primer (qPCR)	DNA oligo, unmodified
miR-221_qPCR_Fw	AGCTACATTGTCTGCTGGGTTTC	miR-221 specific F primer (qPCR)	DNA oligo, unmodified
Nluc2_qPCR_Fw	AGGGTCCCTGTTACAGTGATC	Nluc2 specific F primer (qPCR)	DNA oligo, unmodified
